# Identifying the critical state of complex biological systems by the directed-network rank score method

**DOI:** 10.1093/bioinformatics/btac707

**Published:** 2022-10-25

**Authors:** Jiayuan Zhong, Chongyin Han, Yangkai Wang, Pei Chen, Rui Liu

**Affiliations:** School of Mathematics and Big Data, Foshan University, Foshan 528000, China; School of Mathematics, South China University of Technology, Guangzhou 510640, China; School of Biology and Biological Engineering, South China University of Technology, Guangzhou 510640, China; School of Mathematics, South China University of Technology, Guangzhou 510640, China; School of Mathematics, South China University of Technology, Guangzhou 510640, China; School of Mathematics, South China University of Technology, Guangzhou 510640, China; Pazhou Lab, Guangzhou 510330, China

## Abstract

**Motivation:**

Catastrophic transitions are ubiquitous in the dynamic progression of complex biological systems; that is, a critical transition at which complex systems suddenly shift from one stable state to another occurs. Identifying such a critical point or tipping point is essential for revealing the underlying mechanism of complex biological systems. However, it is difficult to identify the tipping point since few significant differences in the critical state are detected in terms of traditional static measurements.

**Results:**

In this study, by exploring the dynamic changes in gene cooperative effects between the before-transition and critical states, we presented a model-free approach, the directed-network rank score (DNRS), to detect the early-warning signal of critical transition in complex biological systems. The proposed method is applicable to both bulk and single-cell RNA-sequencing (scRNA-seq) data. This computational method was validated by the successful identification of the critical or pre-transition state for both simulated and six real datasets, including three scRNA-seq datasets of embryonic development and three tumor datasets. In addition, the functional and pathway enrichment analyses suggested that the corresponding DNRS signaling biomarkers were involved in key biological processes.

**Availability and implementation:**

The source code is freely available at https://github.com/zhongjiayuan/DNRS.

**Supplementary information:**

Supplementary data are available at *Bioinformatics* online.

## 1 Introduction

**Fig. 1.**
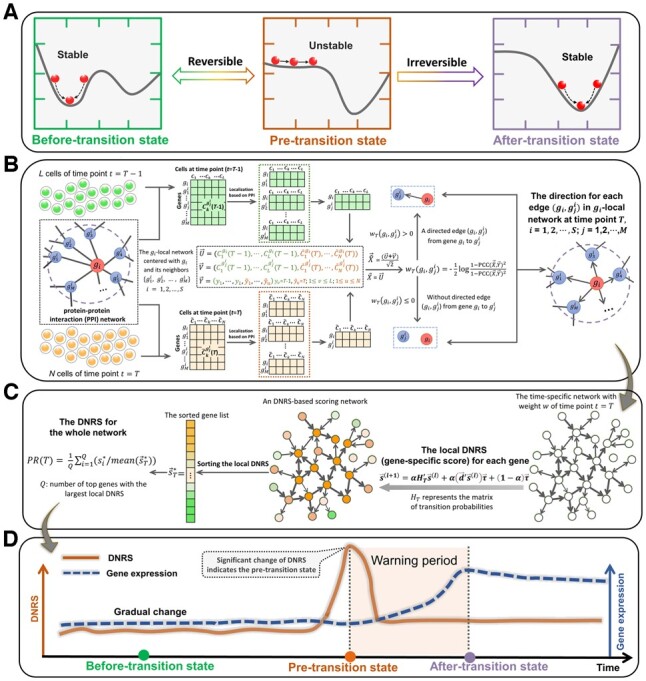
Illustration of the proposed method for detecting the critical transition of complex biological systems based on a modified personalized PageRank. (**A**) The complex biological processes can be roughly divided into three stages/stages, i.e. a before-transition state, a pre-transition state and an after-transition state. (**B**) Given a group of control cells/samples from former time point t=T-1 and a set of case cells/samples derived at time point T, the time-specific directed network of time point T is constructed based on rewiring the protein–protein interaction (PPI) network with direction determination index $w_{T}$. (**C**) The local DNRS is calculated for each node in the time-specific directed network of the time point T based on the personalized PageRank (Eq. 2) and then the DNRS (Eq. 8) is utilized to detect the early-warning signal for the critical transition of a complex biological system. (**D**) During the dynamic progression of a complex biological system, the DNRS remains low when the system is in a before-transition state, while it increases significantly when the system is close to the pre-transition state. Such an abrupt increase in the DNRS indicates the tipping point (or the critical state) of a complex biological system

Many complex systems undergo a critical transition and then switch abruptly to a contrasting state (Scheffer *et al.*, 2009); that is, there exists a so-called tipping point at which a drastic, irreversible and qualitative transition may occur. Detecting such tipping points for general systems, such as socioecological (Pananos *et al.*, 2017), financial (Billio *et al.*, 2016) and climate systems (Boers, 2018), has received extensive attention. In biomedical fields, a similar critical state transition is also observed in a variety of biological processes. For example, some chronic diseases may experience a gradual process that takes several years or even decades before catastrophic deterioration occurs. Cell fate commitment is also regarded as a critical transition of embryonic development, after which there is a drastic change in the cell populations (Zhong *et al.*, 2021). Identifying such a critical transition or tipping point in biological systems plays an essential role in providing insights into the underlying mechanism of disease progression or embryonic development (Guo *et al.*, 2021a; Shi *et al.*, 2021). The time evolution of a complex biological system is usually modeled as a time-dependent non-linear dynamic system (Liu *et al.*, 2020, 2021a), in which the abrupt transition is viewed as the phase shift at a bifurcation point (Scheffer *et al.*, 2009). Therefore, from a dynamic system perspective, a biological process can be broadly divided into three states or stages (Fig. 1A): (i) a before-transition state, a stable state with strong resilience; (ii) a critical/pre-transition state, which is an unstable state just before the onset of qualitative changes and with low resilience and sensitivity to perturbation; and (iii) an after-transition state, another stable state with strong resilience after the qualitative transition. For biological systems, hunting for such a pre-transition state may provide appropriate timing for intervention to prevent or at least prepare for the upcoming catastrophic consequences, such as disease onset or deterioration. The recently proposed applications of a new concept, i.e. the dynamic network biomarker (DNB) (Chen *et al.*, 2012), have been employed to detect the critical transitions in biological systems. Specifically, when the system approaches the tipping point/critical state, a dominant group of strongly fluctuating variables (DNBs) appears and exhibits strong cooperative effects on molecular associations, which could be employed to quantitatively characterize the stability and criticality of a system at the network level (Chen *et al.*, 2022; Guo *et al.*, 2018; Liu *et al.*, 2021b). In the past, many network-based ranking techniques, such as random walk-based methods (Roy *et al.*, 2014; Winter *et al.*, 2012), topological property-based approaches (George *et al.*, 2006; Krauthammer *et al.*, 2004), the Markov random field model (Ma *et al.*, 2007) and the order statistical index (Aerts *et al.*, 2006), have been proposed for the identification of network-based disease biomarkers by exploring the cooperative effects of gene combinations. However, these studies mainly focused on the diagnosis of disease (static biomarker) instead of disease prediction (dynamic biomarker).

Recently, network-based methods from the DNB framework have been developed to address different biological topics, such as the identification of pre-disease states for complex diseases (Liu *et al.*, 2022; Peng *et al.*, 2022), the personalized characterization of diseases (Liu *et al.*, 2017; Zhang *et al.*, 2021) and the discovery of personalized driver genes prioritization (Guo *et al.*, 2021b). From the perspective of gene associative and cooperative effects, we presented a model-free computational method in this study, i.e. the directed-network rank score (DNRS) method, to detect the critical states of complex biological systems and identify key genes involved in related essential biological processes. Specifically, based on the cells/samples from former time point t=T-1, the time-specific directed network at a time point T is constructed based on rewiring the protein–protein interaction (PPI) network with direction determination index $w_{T}$ (Fig. 1B), and then the local DNRS is calculated for each node/gene by using the proposed personalized PageRank (Fig. 1C). The DNRS can be utilized to quantify the dynamic changes in gene cooperative effects of a time-specific directed network. The drastic increase in such DNRS signals an upcoming tipping point or pre-transition state of a biological system (Fig. 1D). Furthermore, at the identified tipping point, a group of biomolecules that exhibit strong cooperative effects on molecular associations are identified as DNRS-signaling biomarkers for further functional analysis. To demonstrate the effectiveness of DNRS in both bulk and single-cell data, the proposed method was applied to a number of datasets, including a simulated dataset, three single-cell datasets of embryonic development and three tumor datasets from The Cancer Genome Atlas (TCGA). Specifically, cell fate commitment was successfully detected in three single-cell datasets of embryonic development, including the differentiation of human embryonic stem cells to definitive endoderm cells (hESC-to-DEC data), the development of epithelial basal cells to the mouse hair follicle (EBC-to-MHF data) and the differentiation of human embryonic stem cells to neurons (hESC-to-neuron data). For three tumor datasets, we identified the pre-transition state before lymph node metastasis (Stage II) for colon adenocarcinoma (COAD), the pre-transition state before the tumor invaded the renal vein (Stage II) for kidney renal clear cell carcinoma (KIRC) and the pre-transition state before distant metastasis (Stage IIIB) for lung adenocarcinoma (LUAD). The identified critical states all agreed with the experimental observations, suggesting the robustness of the DNRS method. In addition, the functional and pathway enrichment analyses revealed that the corresponding DNRS signaling genes were implicated in important biological processes.

## 2 Materials and methods

### 2.1 Theoretical background

The theoretical background of the DNRS approach is based on our recently proposed DNB theory. Generally, from a system perspective, the progression of a complex biological system is described by the dynamic evolution of a high-dimensional non-linear system, where a drastic or qualitative shift in a biological process is regarded as a phase transition at a bifurcation point (Shi *et al.*, 2021). In terms of the DNB theory (Chen *et al.*, 2012; Liu *et al.*, 2019), when the system is in the vicinity of a critical point (bifurcation point), a dominant group of biomolecules defined as the DNB appears, which satisfies the following three properties (Chen *et al.*, 2012):

- The variation in each DNB internal biomolecule rapidly increases;
- The correlation between each pair of DNB internal biomolecules drastically increases;
- The correlation between a DNB internal molecule and any external biomolecule drastically decreases.

From the properties of DNB molecules, as the system is close to the critical point, a group of highly correlated and widely fluctuating variables emerges and exhibits strong cooperative effects on molecular associations, signaling the upcoming critical transition. By exploring the dynamic changes of such a group of dominant variables in molecular associations at the network level, it is possible to predict the qualitative state transition of a system. The proposed DNRS method is designed to quantify the dynamic changes in gene cooperative effects of a time-specific directed network, which captures the criticality of biological systems.

To describe and measure the significant changes in gene cooperative effects at a network level, the PageRank method can be employed to quantify the dynamic changes of molecular associations. Let $G=\left(V,\;E\right)\;$be a directed graph with K nodes. Denote $A=(a_{ij})\in R^{K\times K}$ as the transition matrix of the graph G, and define $d^{\mathrm{out}}\left(i\right)$ as the out-degree of node i. For node i (corresponding to row i), there are two settings corresponding to two different cases: (i) when $d^{\mathrm{out}}\left(i\right)>0$, $a_{\mathrm{ij}}=1/d^{\mathrm{out}}\left(i\right)$ if node i points to node j in graph G, and 0 otherwise; (ii) when $d^{\mathrm{out}}\left(i\right)=0,\;a_{ij}=1$ if j=i, and 0 otherwise. For such graph G, the PageRank vector ${\vec{s}}^{*}$ (the PageRank score of nodes) is obtained by solving the fixed point of the following iteration equation (Langville and Meyer, 2011):

(1) $${\vec{\text{s}}}^{\left(l+1\right)}={\alpha \text{A'}\vec{\text{s}}}^{\left(l\right)}+\frac{\left(1-\alpha \right)}{K},$$

where α is a damping factor that is usually set as 0.85, and the symbol ‘'’ represents the transpose of a matrix. Taking into account the statistical properties of DNB theory, a personalized PageRank is proposed and used in this study as follows:

(2) $${\vec{s}}^{\left(l+1\right)}=\alpha H_{T}^{'}{\vec{s}}^{\left(l\right)}+\alpha \left({\vec{d}}^{'}{\vec{s}}^{\left(l\right)}\right)\vec{\tau }+\left(1-\alpha \right)\vec{\tau },$$

where the matrix $H_{T}$ (as described in Step 2 of the following DNRS algorithm) represents the personalized transition matrix constructed from a time-specific directed network of a time point T. The vector $\vec{d}$ (as presented in Step 3 of the DNRS algorithm) could be used for balancing errors caused by isolated nodes that have no edge pointing to other nodes. Vector $\vec{\tau }$ (as described in Step 4 of the DNRS algorithm) represents a personalized vector that satisfies $\sum_{i=1}^{K}\tau_{i}=1$.

The time-specific directed network at a time point T is constructed based on an information-theoretic scheme (Sun *et al.*, 2019), which provides a direction determination index w (as defined in Eq. 3) to evaluate the combined effect of gene combinations over a single gene from the perspective of mutual information (MI). Specifically, vectors $\vec{U}$ and $\vec{V}$ are denoted as the expression profile of $g_{i}$ and $g_{j}\;$in all cells/samples from two adjacent time points T and T-1, respectively. Vector $\vec{Y}$ is denoted as a binary phenotype label indicating the sampling time point (T or T-1) of each cell, i.e. $y_{k}$= T means that the kth cell/sample is obtained from the sampling time T, while $y_{k}=T-1$ marks the other case. Let vectors $\vec{\hat{X}}$ and $\vec{X}$ be defined as $\vec{\hat{X}}=\;(\vec{U}+\vec{V})/\sqrt{2}$ and $\vec{X}=\vec{U}$, respectively. Then, whether there is a direction for edge ($g_{i},g_{j}$) from gene $g_{i}$ to $g_{j}$ is decided by the direction determination index $w_{i,\;j}$ defined as follows.

(3) $$w_{i,\;j}=\sum_{\hat{x}\in \vec{\hat{X}}}\sum_{y\in \vec{Y}}p(\hat{x},y)\log\frac{p(\hat{x},y)}{p(\hat{x})p(y)}-\sum_{x\in \vec{X}}\sum_{y\in \vec{Y}}p(x,\;y)\log\frac{p(x,\;y)}{p(x)p(y)},$$

where $p(\hat{x},y)$ is the joint probability density function (pdf) of $\vec{\hat{X}}$ and $\vec{Y}$ and $p\left(x,\;y\right)$ is the pdf of $\vec{X}$ and $\vec{Y}$. $p\left(\hat{x}\right),\;p\left(x\right)$ and p(y) represent the marginal pdfs of $\vec{\hat{X}}$, $\vec{X}\;$and $\vec{Y}$, respectively. The positive determination value infers that the integration of gene${\;g}_{j}$ can be considered to be an improvement to the mutual information (MI) of gene $g_{i}$; that is, there is a directed edge ($g_{i},g_{j}$) from gene $g_{i}$ to $g_{j}$ in the time-specific directed network.

If the variables follow Gaussian distribution or binomial distribution, Eq. (3) can be expressed as follows (see Supplementary Information C for derivation).

(4) $$w_{i,\;j}=-\frac{1}{2}\log\frac{1-{\mathrm{PCC}(\vec{\hat{X}},\vec{Y})}^{2}}{1-{\mathrm{PCC}(\vec{X},\vec{Y})}^{2}},$$

where $\mathrm{PCC}(\vec{\hat{X}},\vec{Y})$ represnts the Pearson correlation coefficient (PCC) between the vector $\vec{\hat{X}}$ and $\vec{Y}$, and $\mathrm{PCC}(\vec{X},\vec{Y})$ is the PCC between $\vec{X}$ and $\vec{Y}$. From the properties of the DNB, when the system is close to the vicinity of the critical point, there are much more directed edges appearing for some nodes (i.e. the DNB members) in the time-specific directed network.

### 2.2 Algorithm to identify the critical point based on DNRS

As mentioned above, for a biological system with K genes/variables, the state of the system at each time point can be revealed by the dynamical changes of cooperative effects on molecular associations. The following algorithm is proposed to explore such dynamical changes at a network level and identify the critical state or tipping point.

**[Step 1]** Construct the time-specific directed network $N^{\left(T\right)}$ at each time point t=T (T≥2). Based on the protein–protein interaction (PPI) network and cells/samples from two adjacent time points (e.g. N and L cells/samples at the time point $T\;\mathrm{and}\;\left(T-1\right),\;$respectively), the time-specific directed network $N^{\left(T\right)}$ can be constructed by a direction determination index $w_{i,\;j}$ which is defined as Eq. (4). Specifically, if $w_{i,\;j}$ is greater than zero, there is a directed edge ($g_{i},g_{j}$) from gene $g_{i}$ to $g_{j}$; otherwise, there does not exist a directed edge ($g_{i},g_{j}$). By this way, we construct a time-specific directed network $N^{\left(T\right)}$, where each directed edge ($g_{i},g_{j}$) from gene $g_{i}$ to $g_{j}$ is decided by direction determination index $w_{i,\;j}$.

**[Step 2]** Construct the network’s transition matrix $H_{T}={\left(H_{i,\;j}\right)}_{K\times K}$ based on the time-specific directed network $N^{\left(T\right)}$, where K represents the number of nodes/genes. The matrix element $H_{i,\;j}$ represents the direction determination value $w_{i,\;j}$ if there exists a directed edge from the node i (gene $g_{i}$) to j (gene $g_{j}$), otherwise${\;H}_{i,\;j}$ is set as 0. Then the standardization of matrix $H_{T}$ is as follows:

(5) $$H_{i,\;j}=\frac{w_{i,\;j}}{\sum_{j=1}^{K}w_{i,\;j}}\;\mathrm{if}\;\sum_{j=1}^{K}w_{i,\;j}\neq 0$$

or

(6) Hi, j=0 (j≠i)1 (j=i) if ∑j=1Kwi, j=0

**[Step 3]** Build a K-dimensional vector $\vec{d},\;$which can be used for balancing errors caused by isolated nodes which have no edge pointing to other nodes. Its elements can be defined as the following criteria.

(7) di=1 ∑j=1Kwi, j=0 0 ∑j=1Kwi, j≠0  i=1, 2, …,K

**[Step 4]** Build a K-dimensional personalized vector$\;\vec{\tau },$ where the element $\tau_{i}$ represents the standard deviation of node i (gene $g_{i}$) based on the gene expressions of N cells at sampling time point T. The normalized vector $\vec{\tau }$ can be obtained by setting each of its element as $\tau_{i}=\tau_{i}/\sum_{i=1}^{K}\tau_{i}$.

**[Step 5]** Calculate the PageRank vector$\;{\vec{s}}_{T}^{*}=(s_{1}^{*},\ldots ,s_{K}^{*})$ (presented as Eq. 2) for the time-specific directed network $N^{\left(T\right)}$, that is, the local DNRS (gene-specific local DNRS) is calculated for each gene of the time-specific directed network $N^{\left(T\right)}$. Then the DNRS for the whole network can be obtained from the following formula:

(8) $$\mathrm{PR}\left(T\right)=\;\frac{1}{Q}\sum_{i=1}^{Q}\left(\frac{s_{i}^{*}}{\mathrm{mean}\left({\vec{s}}_{T}^{*}\right)}\right),$$

where Q is the number of the top 5% genes with the largest local DNRS and the symbol $\mathrm{mean}({\vec{s}}_{T}^{*})$ represents the mean of the PageRank vector ${\vec{s}}_{T}^{*}$.

**[Step 6]** The critical point is identified by the one-sample *t* test (Rochon and Kieser, 2011), which is employed to. To analyze how well the DNRS recapitulates the abrupt transition, the one-sample *t* test index Z is used to determine whether value x is significantly different from the mean of n-dimensional vector $X=(x_{1},x_{2,\ldots ,}{\;x}_{n})$, namely,

(9) $$Z=\frac{\mathrm{mean}(X)-x}{s/\sqrt{n}},$$

where mean(X) represents the mean of vector X and the s is the standard deviation of vector X. The *P*-value related to index Z is derived from the t-distribution to assess the statistical difference between mean(X) and x. There is a statistically significant difference between mean(X) and x if P<0.05. In this study, the time point t=T is viewed as a critical point if PR(t) satisfies two criteria: (i) PR(t)> PR(t-1); (ii) PR(t) is statistically different (P<0.05) from the prior values (also see Supplementary Information D).

Based on the DNB theory, the DNB biomolecules exhibit strong fluctuations in a synchronized and collective manner when a biological system is close to the critical point (Chen *et al.*, 2012). Thus, when the system approaches the pre-transition state, some key biomolecules within the time-specific directed network $N^{\left(T\right)}$ yield significant dynamic changes in molecular cooperative effects or gene associations, which lead to a significant increase of PR(T), thus implying the imminent critical transition.

### 2.3 Data preprocessing and functional analysis

The DNRS method has been applied to six high-throughput sequencing datasets, including EBC-to-MHF data (ID: GSE147372) (Morita *et al.*, 2021), hESC-to-DEC data (ID: GSE75748) (Chu *et al.*, 2016) and hESC-to-neuron data (ID: GSE86977) (Yao *et al.*, 2017) from the NCBI Gene Expression Omnibus (GEO) database (http://www.ncbi.nlm.nih.gov/geo) and COAD, KIRC and LUAD from TCGA database (http://cancergenome.nih.gov). For all sequencing datasets, genes without the corresponding NCBI Entrez gene symbol were discarded. For the gene mapped with multiple probes, the mean value was taken as its expression. The tumor datasets included both tumor and adjacent non-tumor samples. Based on the corresponding clinical information of TCGA, the tumor samples were classified into several cancer stages. Other information of the datasets is given in Supplementary Information B.

The functional annotations were obtained according to the NCBI Gene database (http://www.ncbi.nlm.nih.gov/gene). The enrichment analysis was performed based on DAVID (Huang *et al.*, 2009), Metascape (Zhou *et al.*, 2019) and the ClusterProfiler package (Yu *et al.*, 2012). All pathway information was obtained from the Kyoto Encyclopedia of Genes and Genomes (KEGG) (https://www.kegg.jp/).

## 3 Results

The definition and algorithm of the DNRS were presented in the above section. To illustrate how DNRS works, we first applied it to a simulated dataset, and then to six real-world datasets, i.e. single-cell sequencing datasets including EBC-to-MHF data, hESC-to-DEC data, hESC-to-neuron data and TCGA bulk datasets including COAD, KIRC and LUAD. The detailed description of these datasets is given in Supplementary Information B. For all datasets, the proposed method successfully identified the pre-transition state or detected the early-warning signals of critical transition into an irreversible after-transition state, which validated the effectiveness of our method in quantifying the critical point just before the critical transition into the after-transition state.

### 3.1 Validation based on numerical simulation

An 18-node regulatory network (Supplementary Fig. S1) is employed to demonstrate the performance of the proposed method. Such regulatory network represented in Michaelis-Menten form is described by stochastic differential equations Eq. (S1) provided in Supplementary Information A and is classically used to study gene regulatory activities such as transcription, translation and non-linear biological processes (Chen *et al.*, 2009). In Supplementary Eq. (S1), with parameter s varying from -0.5 to 0.15 and s=0 as the bifurcation point, a numerical simulation dataset was generated from the network.

**Fig. 2.**
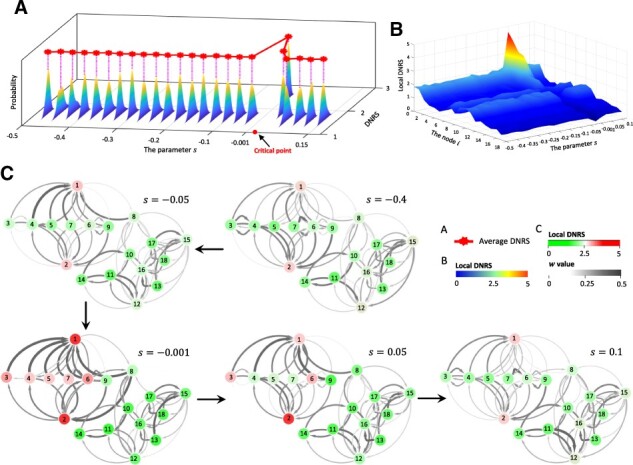
Validation of the DNRS approach based on numerical simulation. (**A**) We presented the evolution of the mean values of DNRS (defined in Eq. (8)) based on 50 simulated trials. It is clear that DNRS abruptly increases near the critical point s=0. (**B**) The landscape evolution of local DNRS is presented for different nodes. Notably, the local DNRS of DNB members exhibits a sharp increase when the system is close to the bifurcation point (s=0). (**C**) From the dynamic evolution of the regulatory network, it is seen that an obvious change in the structure of the subnetwork composed of DNB members appears near the tipping point s=0

As shown in Figure 2A, the DNRS increases rapidly when the dynamic system is close to a special parametric value s=0 (the bifurcation point). Moreover, to assess the robustness of our approach, a set of samples was generated with additive white noise. The evolution of the mean values of DNRS (the red curve in Fig. 2A) also stably provides the early-warning signal of the critical point, which illustrates that the DNRS is robust against sample noise. In addition, we have analyzed the stability and robustness of the proposed method under different levels of data noise (Supplementary Fig. S2). To exhibit the distinct dynamics of the system between the before-transition and pre-transition state, we presented the landscape evolution of local DNRS of different nodes (Fig. 2B). It is seen from such a landscape that the local DNRS of the so-called DNB members (see Supplementary Information A for details) exhibit a sharp increase in the pre-transition state (s=-0.001). In addition, the dynamic evolution of the regulatory network is shown in Figure 2C, where an obvious change in the structure of the subnetwork composed of DNB members appears near the bifurcation point s=0, signaling the upcoming state transition at the network level. In other words, when the system approaches the tipping point, DNB members yield a distinct change in cooperative effects on molecular associations, resulting in a dramatic increase in the corresponding local DNRS. Such a critical phenomenon can be accurately detected by the proposed approach, which demonstrates the effectiveness of DNRS in detecting the early warning signal of critical transition. The detailed description of the dynamic system is given in Supplementary Information A.

### 3.2 Identifying critical transitions for both embryonic development and cancers

**Fig. 3.**
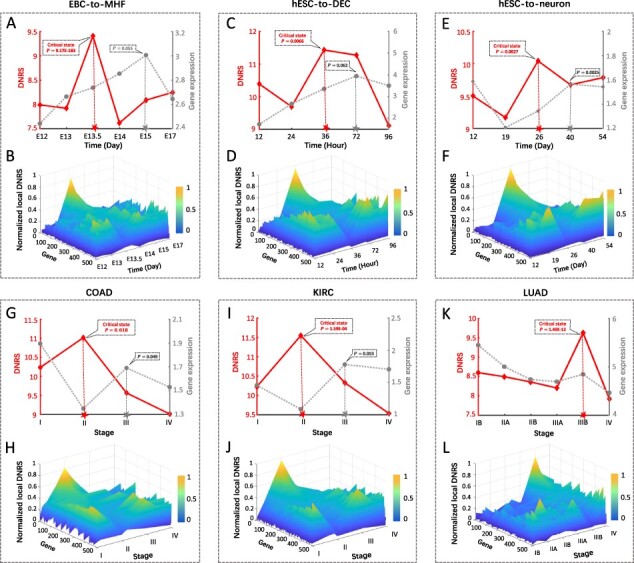
The application of the DNRS method in both embryonic development and tumor diseases. The performance of dynamic changes between DNRS and the mean gene expression for six biological datasets: (**A**) EBC-to-MHF data, (**C**) hESC-to-DEC data, (**E**) hESC-to-neuron data, (**G**) COAD, (**I**) KIRC and (**K**) LUAD. The landscape of local DNRS illustrates the dynamic evolution of signaling and non-signaling genes for these six real-world datasets: (**B**) EBC-to-MHF data, (**D**) hESC-to-DEC data, (**F**) hESC-to-neuron data, (**H**) COAD, (**J**) KIRC and (**L**) LUAD

To illustrate how the DNRS works on real datasets, the proposed method was applied to three scRNA-seq datasets of embryonic development (EBC-to-MHF data, hESC-to-DEC data and hESC-to-neuron data) and three tumor datasets (COAD, KIRC and LUAD). The DNRS was calculated for each time point based on Eq. (8) and then taken to detect any possible critical state. For these datasets, the sharp increase in DNRS successfully indicated an upcoming critical transition just before the irreversible after-transition state (the red curve in Fig. 3A, C, E, G, I and K), which validated the effectiveness of the proposed method. At each identified critical point, the top 5% of genes with the largest local DNRS were selected as the signaling genes, which could be regarded as a gene set containing the dynamic network biomarker.

When applied to three scRNA-seq datasets, DNRS detects the early warning signal of cell fate commitment during embryonic development. For the EBC-to-MHF data, as shown in the red curve in Figure 3A, the DNRS sharply increased at embryonic point 13.5 (E13.5) (P=9.173E-183), after which it was observed that epithelial basal cells were induced into hair follicle stem cells (HFSCs) (Morita *et al.*, 2021). As shown by the gray curve in Figure 3A, the mean gene expression of the differentially expressed genes (DEGs) showed dynamic changes at E15 (P= 0.055), failed to provide an early-warning signal for cell fate transition. In addition, the landscape of the local DNRS for signaling and non-signaling genes is illustrated in Figure 3B, where a group of genes (signaling genes) exhibits a significant increase in local DNRS at E13.5. The hESC-to-DEC data are presented by the red curve in Figure 3C, the drastic transition (P=0.0066) of DNRS from 24 h to 36 h appears and reaches its peak at 36 h, indicating a commitment to a definitive endoderm fate occurred at 72 h (Chu *et al.*, 2016). The dynamic change (P= 0.062) in the mean expression of DEGs appear at 72 h (the gray curve in Fig. 3C). Moreover, it is seen from Figure 3D that the peak of local DNRS for signaling genes appears at 36 h. When applied to hESC-to-neuron data, there was an abrupt increase (P=0.0027) in DNRS from Day 19 to Day 26 (the red curve in Fig. 3E), signaling the upcoming differentiation of progenitor cells into neuronal cells at Day 40 (Yao *et al.*, 2017). The gray curve in Figure 3E shows that the mean expression of DEGs exhibited a significant difference (P=0.0025) at Day 40, failing to provide timely early-warning signals for the cell fate transition. In addition, Figure 3F shows that a significant increase in local DNRS of signaling genes occurs on Day 26.

For three tumor datasets, the proposed method also identified the critical point just before a catastrophic transition to the worsening of diseases. As shown by the red curve in Figure 3G, for COAD, a significant change (P=0.018) in DNRS was detected around Stage II, suggesting lymph node metastasis and tumor invasion of other adjacent organs in Stage III (Hari *et al.*, 2013). The gray curve in Figure 3G shows that a significant increase (P=0.049) in mean expression of DEGs occurs in Stage III and fails to detect the pre-deterioration stage. Additionally, the landscape of local DNRS for signaling and non-signaling genes is presented in Figure 3H, from which it is seen that a significant increase in the local DNRS of signaling genes occurs in Stage II. When applied to KIRC, it is seen from Figure 3I that the peak of DNRS (P=1.592E-04) appears in Stage II, after which the lipid levels around the kidney increases rapidly, and then the tumor invades the renal vein (Su and Shahriyari, 2020). As presented in gray in Figure 3I, dynamic changes (P=0.053) in the mean gene expression were detected in Stage III and therefore failed to provide timely early-warning signals of the cell fate transition. Furthermore, Figure 3J indicates that the local DNRS of signaling genes from Stage I to Stage II abruptly increases and reaches its peak in Stage II, revealing the imminent critical transition after Stage II. For LUAD, as illustrated in the red curve in Figure 3K, the DNRS score in Stage IIIB significantly increased (P=1.488E-12), indicating an upcoming critical transition in Stage IV; that is, Stage IV was characterized by a distant metastasis process, in which the tumor cells invaded distant tissues or organs (Chiang and Massagué, 2008). However, in terms of mean gene expression, there was little significant difference among the six time points (the gray curve in Fig. 3K). In addition, Figure 3L demonstrates that the signaling genes exhibit an abrupt increase in local DNRS around Stage IIIB. The identified pre-deterioration stage are actually closely related to prognosis based on Kaplan–Meier log-rank analysis (see Supplementary Information E for details), that is, the survival times based on samples from the before-transition and after-transition stages are significantly different (P<0.05) as shown in Supplementary Figures S4 and S5.

### 3.3 Inferring the dynamical evolution of the regulatory networks for signaling genes

**Fig. 4.**
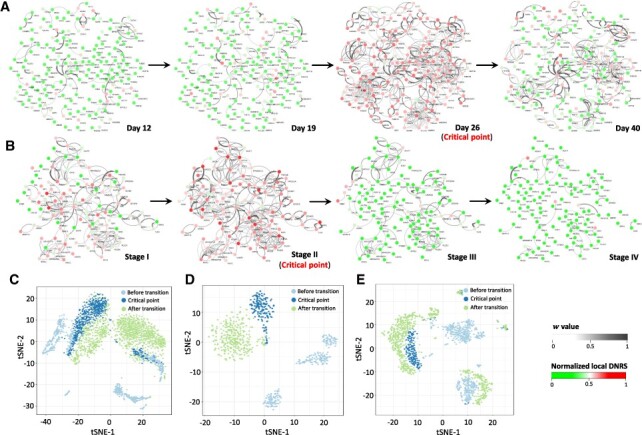
The dynamic evolution of the regulatory networks for signaling genes. The signaling genes were mapped to the PPI network, where the maximal connected subgraph was extracted to study the dynamic evolution of the regulatory network for signaling genes. Each node represents a gene with the local DNRS, and each edge represents the regulation with the direction determination value w. (**A**) The dynamic evolution of signaling gene networks for hESC-to-neuron data. (**B**) The dynamic evolution of signaling gene networks for the TCGA-COAD dataset. Based on the expression levels of signaling genes (the top 5% of genes with the highest local DNRS at the tipping point), t-SNE was employed to cluster cells for (**C**) hESC-to-neuron data, (**D**) hESC-to-DEC data and (**E**) EBC-to-MHF data

At the identified tipping point, the top 5% of genes with the largest local DNRS were selected as the signaling genes for further analyses of function and biological process. These signaling genes can be viewed as DNBs and may play key roles in triggering the critical transitions of biological systems. The signaling genes were mapped to the PPI network, where the maximal connected subgraph was extracted to study the dynamic evolution of the regulatory network of signaling genes. For hESC-to-neuron data, an obvious change occurs in the network structure on Day 26 (Fig. 4A), indicating the cell fate transition of progenitor cells into neuronal cells on Day 40 (Yao *et al.*, 2017). The dynamic evolution of the regulatory network across all 5 time points is given in Supplementary Figure S6A. The dynamic evolution of the regulatory network for hESC-to-DEC data can be seen in Supplementary Figure S6B. When applied to the TCGA-COAD dataset, there was a notable change in the network structure at Stage II (Fig. 4B), implying the imminent critical transition, that is, there was lymph node metastasis and tumor invasion of adjacent organs after Stage II (Hari *et al.*, 2013). In addition, as shown in Figure 4C–E, for three datasets of embryonic development, the expressions of signaling genes effectively distinguish the state of cells before and after critical transition by using t-distributed stochastic neighbor embedding (t-SNE) (Van der Maaten and Hinton, 2008).

### 3.4 The regulation mechanisms underlying embryonic development

**Fig. 5.**
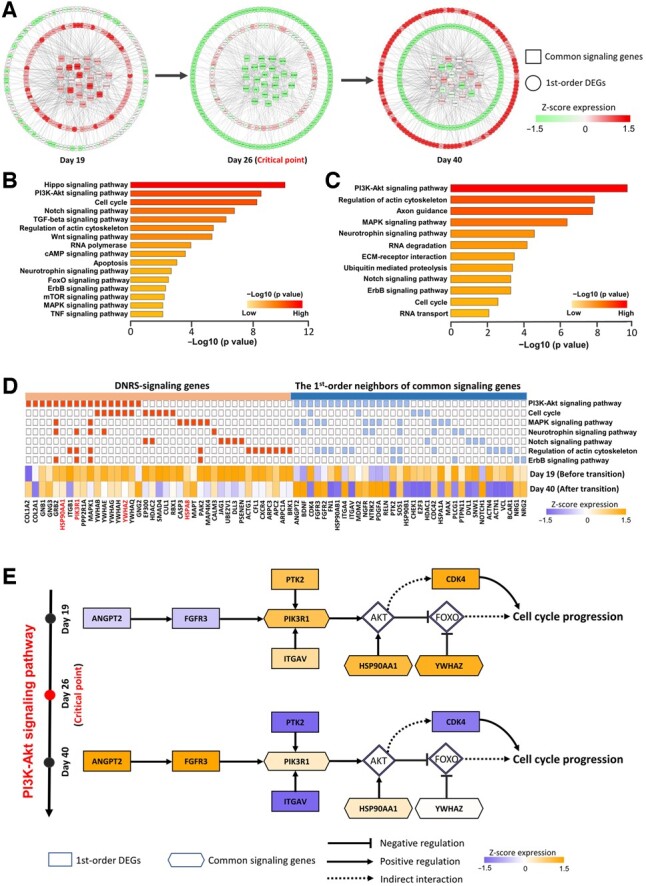
The potential mechanisms underlying the functional associations during embryonic development. (**A**) Dynamic evolution of the PPI subnetwork of common signaling genes (CSGs) consists of 24 CSGs and their 210 first-order DEG neighbors for the hESC-to-neuron process. (**B**) The pathway enrichment analysis for the DNRS signaling genes from the hESC-to-neuron data. (**C**) The pathway enrichment analysis for the first-order DEG neighbors of CSGs. (**D**) The key common pathways are shared between DNRS-signaling genes and the first-order DEG neighbors of CSGs. (**E**) The underlying molecular mechanism is revealed by the functional analysis of CSGs and their first-order DEG neighbors

For two scRNA-seq datasets (hESC-to-DEC and hESC-to-neuron data) from human embryonic development, 24 common signaling genes (CSGs) were shared between these two datasets (Supplementary Fig. S7A). To explore the regulatory mechanisms underlying embryonic development at the network level, we analyze the PPI subnetwork of CSGs, which is composed of the CSGs and their first-order DEG neighbors from the PPI network. The first-order DEG neighbors are genes satisfying: (i) they are the first-order neighbors of CSGs in the PPI network; and (ii) they are differentially expressed; that is, there are significant differences (P<0.05) between gene expressions before and after the identified critical point. As shown in Figure 5A, this subnetwork contains 24 CSGs and 210 first-order DEG neighbors in the hESC-to-neuron process. It is clear that there is a major shift in gene expression for the network after the critical point, that is, gene expression undergoes a significant change either from low to high, or vice versa. Moreover, KEGG pathway enrichment analysis was carried out to investigate the potential mechanisms underlying the functional associations between DNRS-signaling genes and the first-order DEG neighbors of CSGs (Fig. 5B–E). It is seen from Figure 5B–D that the main enriched pathways are closely related to embryonic development. For example, the PI3K/Akt pathway is an intracellular signaling pathway during embryonic development that often induces positive impacts on cell proliferation, differentiation and growth (Hao *et al.*, 2019). The cell cycle pathway plays an important role in the regulation of cell proliferation and differentiation (Hydbring *et al.*, 2016). The MAPK signaling pathway regulates multiple cellular processes, including cell proliferation, differentiation and development (Takahara *et al.*, 2019).

In the PI3K/AKT pathway, the core effector AKT induces a large number of downstream biological effects, such as cell survival, migration and proliferation; therefore, AKT activity is usually tightly regulated (Manning and Cantley, 2007). As shown in Figure 5E, the underlying signaling mechanism of the PI3K/AKT pathway was revealed by the functional analysis of CSGs and their first-order DEG neighbors. The first-order DEG neighbors, such as *ANGPT2* and *FGFR3*, are upstream growth factor signaling genes, the expression of which rises sharply after crossing the critical point, and this dramatic change in gene expression suggests that the critical point detected by the DNRS is possibly the important period for initiation of these signals. The delayed change in the expression of the important coordinating gene *PIK3R1* (an identified CSG) suggests that it receives signals from upstream growth factors and functions to promote cell cycle progression during a specific critical period, which also indicates the precision of the intracellular transcriptional regulatory network during embryonic development. In addition, the first-order DEG neighbor *PTK2* usually acts as an early cascade factor to promote PI3K/AKT signaling and expansion. In addition, two CSGs, *HSP90AA1* and *YWHAZ*, are coregulators that promote cell cycle maintenance by repressing the expression of FOXO transcription factors (Gan *et al*., 2009). These genes showed significant changes in expressions after the critical point and uniformly exhibited a facilitative effect on cell cycle progression. Overall, the synergy of CSGs and their first-order DEG neighbors may reveal the underlying signaling mechanisms involved in cell cycle progression.

### 3.5 Functional analysis of the CSGs among three cancers

**Fig. 6.**
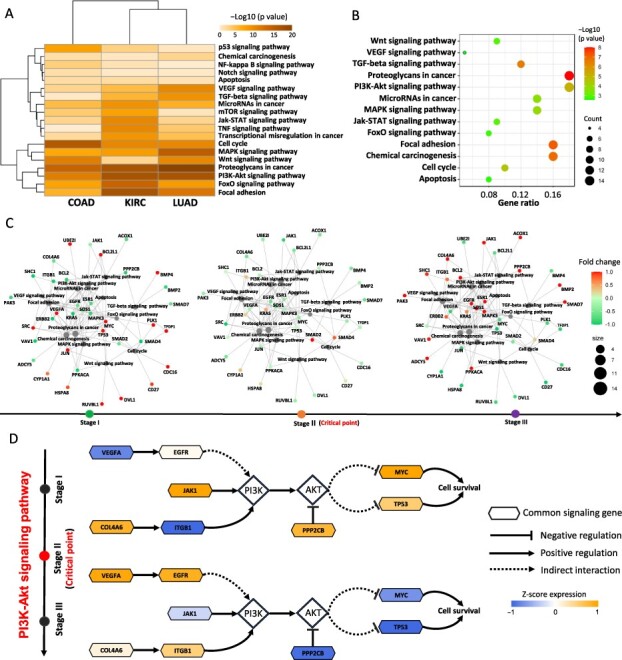
Functional analysis of common DNRS signaling genes. (**A**) KEGG enrichment analysis for the signaling genes from COAD, KIRC and LUAD. (**B**) KEGG enrichment analysis for CSGs among these three cancers. Functional enrichment analysis demonstrated that the CSGs were mainly enriched in cancer-related pathways. (**C**) In the TCGA-COAD dataset, the expression patterns of these common genes involved in different cancer-related pathways were turnover before and after the critical point. (**D**) For COAD, the CSGs enriched in the PI3K/Akt pathway show different regulatory patterns at the critical point before and after lymph node metastasis and play a significant role in the progression of cancer

As shown in Figure 6A, the KEGG enrichment analysis illustrated that signaling genes (top 5% genes with the highest local DNRS) in these cancer datasets were mainly enriched in cancer-related pathways, such as PI3K-Akt signaling pathway, Proteoglycans in cancer, FoxO signaling pathway, MAPK signaling pathway, and Wnt signaling pathway. Moreover, there were not only many intersections across the signaling genes in different cancers, but there existed close functional relationships among them (Supplementary Fig. S7B). To further reveal the genes that might stimulate tumor progression across the different types of cancer, the functional enrichment analysis was performed for 90 CSGs among these three cancers (Supplementary Fig. S7C). Figure 6B shows that these CSGs were enriched in the PI3K-Akt signaling pathway, proteoglycans in cancer and other cancer-related pathways (Supplementary Fig. S7D). Besides, some CSGs have been reported to be closely associated with tumor progression and metastasis (Supplementary Table S1), suggesting that these genes may play important roles in the corresponding biological processes. Moreover, for the TCGA-COAD dataset, we also found that the expression patterns of the common genes involved in different cancer-related pathways switched before and after critical points (Fig. 6C). In colorectal cancer progression, the critical point appears in clinical Stage II, a stage that usually distinguishes the occurrence of lymph node metastasis of colorectal cancer (Lan *et al.*, 2012). Figure 6D uncovers the CSGs enriched in the PI3K/Akt pathway that show different regulatory patterns before and after lymph node metastasis and play important roles in the progression of cancer. Specifically, the genes *MYC* and *TP53*, which are closely associated with cell survival, show increased expression before the critical period, and these genes are thought to be directly related to apoptotic function (Kennedy *et al.*, 1997; Yoon *et al.*, 2015), suggesting that antagonism of cancer cells to apoptosis is completed before the critical state and, more specifically, before tissue infiltration or lymph node metastasis. Furthermore, the expression products of *VEGFA* and *EGFR* are often regarded to promote tumor tissue angiogenesis and thus tumor infiltration and metastasis via the PI3K-AKT pathway (Chen *et al.*, 2014). In Stage III after the critical point, the expression of *VEGFA* and *EGFR* genes increased substantially, which was largely consistent with the subsequent clinical manifestations, and the presence of a large number of angiogenesis-promoting factors drove the distal metastatic foci of colorectal cancer itineraries. In general, the above results suggest that in cancer progression, maintaining cell survival is as a priority for cancer cells before further malignant features such as cell proliferation and tissue infiltration. The presence of stage inconsistencies in the emergence of cancer-associated abnormal cellular functions may be the key to stage-specific precision therapy.

## 4 Discussion

It is important to hunt for the critical state of complex biological systems, such as the pre-deterioration stage of tumor disease and cell fate commitment during embryonic development. Detecting early warning signals for critical transition just before disease deterioration may provide appropriate timing to prevent or at least prepare for catastrophic deterioration. Understanding the cell fate decision may enable the construction of individual-specific disease models and the design of specific therapies for complex diseases relevant to cell differentiation (Peltier and Schaffer, 2010). However, it is usually challenging to identify the critical transition of complex biological systems since there is little change in the system state before reaching the tipping point. In addition, real biological datasets are sometimes too noisy to characterize the dynamics of biological processes.

In this study, different from traditional methods based on the information of differential expressions, we developed a computational method to explore the dynamic changes in cooperative effects on molecular associations, thus signaling an upcoming critical transition when the complex biological system is close to the tipping point. The proposed DNRS has been successfully applied to both a numerical dataset (Fig. 2) and six real biological datasets (Fig. 3). Specifically, the abrupt increase in the DNRS demonstrates the tipping point (E13.5) of the EBC-to-MHF process before differentiation into the mouse hair follicle, the tipping point (36 h) of the hESC-to-DEC process before differentiation into the definitive endoderm, the tipping point (Day 26) of the hESC-to-neuron process before differentiation into neuronal cells, the critical state (Stage II) of COAD before lymph node metastasis, the critical state (Stage II) of KIRC before the tumor invades the renal vein, and the critical state (Stage IIIB) of LUAD before distant metastasis. In addition, functional enrichment analysis revealed that the common DNRS signaling genes for three cancer datasets and two human embryonic differentiation datasets are involved in significant biological processes or pathways (Figs 5 and 6). However, DNRS may not perform well if there are too few samples at a specific time point. In addition, the time-specific directed network is constructed based on a priori knowledge-based PPI network.

To summarize, there are the following advantages of the proposed method. First, compared with the classical DNB, the DNRS is more sensitive to critical signals (see Supplementary Information H for details). Second, compared to the traditional biomarkers that are used for the detection of the after-transition state based on the information of differential expressions, the DNRS method can detect early-warning signals for the pre-transition state just before the catastrophic transition by exploring the dynamic changes of in cooperative effects on molecular associations. Third, it is noteworthy that the DNRS is model-free and different from conventional machine learning models; that is, it requires neither feature selection nor parameter training. Together with the dynamic prediction method (Chen *et al.*, 2020), the DNRS may not only detect the criticality of a system, but reveal the dynamic change in molecular associations that may drive the system into an irreversible state transition.

## Funding

This work was supported by National Natural Science Foundation of China [12026608, 62172164, 12271180 and 12131020]; Guangdong Basic and Applied Basic Research Foundation [2019B151502062]; and Guangdong Provincial Key Laboratory of Human Digital Twin [2022B1212010004].

*Conflict of Interest*: none declared.

## Supplementary Material

btac707_Supplementary_Data

## Data Availability

EBC-to-MHF data (ID: GSE147372), hESC-to-DEC data (ID: GSE75748), and hESC-to-neuron data (ID: GSE86977) are accessible from the NCBI Gene Expression Omnibus (GEO) database (http://www.ncbi.nlm.nih.gov/geo). Colon adenocarcinoma (COAD), kidney renal clear cell carcinoma (KIRC), and lung adenocarcinoma (LUAD) are accessible from the cancer genome atlas (TCGA) database (http://cancergenome.nih.gov).
