# Supplementary material for: Identifying the critical state of complex biological systems by the directed-network rank score method
Source: Bioinformatics. 2022 Oct 25;38(24):5398–405. doi: 10.1093/bioinformatics/btac707 (PMC9750123; doi:10.1093/bioinformatics/btac707)
Supplement: btac707_Supplementary_Data [file btac707_supplementary_data.pdf]

Supplementary Information: Identifying the critical state of complex biological systems by the directed-network rank score method

Contents

A. Detailed description of dynamical system for numerical simulation S2

B. Description of the six datasets S7

C. The derivation for direction discrimination index w S8

D. Description of identifying the critical point by the one-sample t-test S9

E. Validating the identified critical stage by the kaplan-Meier log rank analysis S9

F. The dynamical evolution of the regulatory networks for signaling genes S11

G. Common signaling genes shared between different datasets S12

H. Several improvements of DNRS compared with the classical DNB method S14

I. Analysis of the different settings of the adjustable parameter Q S15

A. Detailed description of dynamical system for numerical simulation

To demonstrate the performance of our proposed DNRS method, the numerical simulation was performed based on an 18-node regulatory network (Figure S1). Such regulatory network is usually applied is applied in the study of various biological processes, including transcription, translation, and diffusion [1,2]. The 18-node regulatory network represented in Michaelis-Menten form can be described by following 18 differential equations.

$$
\begin{cases}
\frac{dz_1(t)}{dt} = \frac{(8-4|s|)z_2(t)}{15(1+z_2(t))} - \frac{(4+4|s|)}{15} z_1(t) + \zeta_1(t) \\
\frac{dz_2(t)}{dt} = \frac{(4-2|s|)z_1(t)}{15(1+z_1(t))} - \frac{(8+2|s|)z_2(t)}{15(1+z_2(t))} + \zeta_2(t) \\
\frac{dz_3(t)}{dt} = \frac{(4|s|-10)}{15} + \frac{(5-2|s|)}{15(1+z_1(t))} + \frac{(5-2|s|)}{15(1+z_2(t))} - z_3(t) + \zeta_3(t) \\
\frac{dz_4(t)}{dt} = \frac{(12-4|s|)}{15} + \frac{(2|s|-6)}{15(1+z_1(t))} + \frac{(2|s|-6)}{15(1+z_2(t))} - \frac{6}{5} z_4(t) + \zeta_4(t) \\
\frac{dz_5(t)}{dt} = \frac{(4|s|-14)}{15} + \frac{(7-2|s|)}{15(1+z_1(t))} + \frac{(7-2|s|)}{15(1+z_2(t))} - \frac{7}{5} z_5(t) + \zeta_5(t) \\
\frac{dz_6(t)}{dt} = \frac{(4|s|-16)}{15} + \frac{(8-2|s|)}{15(1+z_1(t))} + \frac{(8-2|s|)}{15(1+z_2(t))} - \frac{8}{5} z_6(t) + \zeta_6(t) \\
\frac{dz_7(t)}{dt} = \frac{(18-4|s|)}{15} + \frac{(2|s|-9)}{15(1+z_1(t))} + \frac{(2|s|-9)}{15(1+z_2(t))} - \frac{9}{5} z_7(t) + \zeta_7(t) \\
\frac{dz_8(t)}{dt} = -\frac{2z_1(t)}{15(1+z_1(t))} - \frac{2z_2(t)}{15(1+z_2(t))} - \frac{2z_6(t)}{5(1+z_6(t))} + \frac{2z_{10}(t)}{5(1+z_{10}(t))} + \frac{3z_{12}(t)}{5(1+z_{12}(t))} \\
\quad + \frac{z_{15}(t)}{5(1+z_{15}(t))} - \frac{z_{16}(t)}{5(1+z_{16}(t))} - 2z_8(t) + \zeta_8(t) \\
\frac{dz_9(t)}{dt} = -\frac{z_1(t)}{5(1+z_1(t))} - \frac{z_2(t)}{5(1+z_2(t))} - \frac{3z_6(t)}{5(1+z_6(t))} - \frac{11}{5} z_9(t) + \zeta_9(t) \\
\frac{dz_{10}(t)}{dt} = \frac{3z_{12}(t)}{5(1+z_{12}(t))} - \frac{12}{5} z_{10}(t) + \zeta_{10}(t) \\
\frac{dz_{11}(t)}{dt} = \frac{z_{12}(t)}{4(1+z_{12}(t))} - \frac{13}{5} z_{11}(t) + \zeta_{11}(t) \\
\frac{dz_{12}(t)}{dt} = \frac{2z_{15}(t)}{5(1+z_{15}(t))} - \frac{2z_{16}(t)}{5(1+z_{16}(t))} - \frac{14}{5} z_{12}(t) + \zeta_{12}(t) \\
\frac{dz_{13}(t)}{dt} = -\frac{z_{15}(t)}{5(1+z_{15}(t))} - \frac{19z_{16}(t)}{5(1+z_{16}(t))} - 5z_{13}(t) + \zeta_{13}(t) \\
\frac{dz_{14}(t)}{dt} = -\frac{4z_{10}(t)}{5(1+z_{10}(t))} - \frac{4z_{12}(t)}{5(1+z_{12}(t))} - \frac{16}{5} z_{14}(t) + \zeta_{14}(t) \\
\frac{dz_{15}(t)}{dt} = \frac{z_{16}(t)}{10(1+z_{16}(t))} - \frac{7}{2} z_{15}(t) + \zeta_{15}(t) \\
\frac{dz_{16}(t)}{dt} = \frac{z_{15}(t)}{10(1+z_{15}(t))} - \frac{7}{2} z_{16}(t) + \zeta_{16}(t) \\
\frac{dz_{17}(t)}{dt} = -\frac{z_{15}(t)}{10(1+z_{15}(t))} + \frac{z_{16}(t)}{10(1+z_{16}(t))} - \frac{19}{5} z_{17}(t) + \zeta_{17}(t) \\
\frac{dz_{18}(t)}{dt} = -\frac{z_{15}(t)}{10(1+z_{15}(t))} + \frac{z_{16}(t)}{10(1+z_{16}(t))} - \frac{z_{17}(t)}{5(1+z_{17}(t))} - 4z_{18}(t) + \zeta_{18}(t)
\end{cases} \qquad (S1)
$$

where s stands for a scalar control parameter and $\varsigma_i(t)$ (i = 1, 2, ..., 18) represents Gaussian

noises with zero means and covariances $k_{ij} = Cov(\varsigma_i, \varsigma_j)$. $z_i(t)$ (*i* = 1, 2, ..., 18) is denoted as mRNA-i concentrations. In Eq.(S1), the degradation rates of mRNAs is presented as $\mathrm{R}=(\frac{(4+4|s|)}{15},\frac{(8+2|s|)}{15},1,\frac{6}{5},\frac{7}{5},\frac{8}{5},\frac{9}{5},2,\frac{11}{5},\frac{12}{5},\frac{13}{10},\frac{14}{10},5,\frac{16}{10},\frac{7}{2},\frac{7}{2},\frac{19}{5},4)$.

$\overline{Z} = (\overline{z_1}, \overline{z_2}, \overline{z_3}, ..., \overline{z_{18}}) = (0,0,0,...,0)$ is stable equilibrium point of the dynamic system Eq.(S1) . On the basis of the Euler scheme [3], the system Eq.(S1) can be transformed into the following difference equations $Z(k+1) = f(Z(k), S)$ with a small-time interval Δt.

$$
\begin{cases}
z_1(k+1) = z_1(k) + \left[\frac{(8-4|s|)z_2(t)}{15(1+z_2(t))} - \frac{(4+4|s|)}{15} z_1(t) + \zeta_1(t) \right] \Delta t \\
z_2(k+1) = z_2(k) + \left[\frac{(4-2|s|)z_1(t)}{15(1+z_1(t))} - \frac{(8+2|s|)z_2(t)}{15(1+z_2(t))} + \zeta_2(t) \right] \Delta t \\
z_3(k+1) = z_3(k) + \left[\frac{(4|s|-10)}{15} + \frac{(5-2|s|)}{15(1+z_1(t))} + \frac{(5-2|s|)}{15(1+z_2(t))} - z_3(t) + \zeta_3(t) \right] \Delta t \\
z_4(k+1) = z_4(k) + \left[\frac{(12-4|s|)}{15} + \frac{(2|s|-6)}{15(1+z_1(t))} + \frac{(2|s|-6)}{15(1+z_2(t))} - \frac{6}{5} z_4(t) + \zeta_4(t) \right] \Delta t \\
z_5(k+1) = z_5(k) + \left[\frac{(4|s|-14)}{15} + \frac{(7-2|s|)}{15(1+z_1(t))} + \frac{(7-2|s|)}{15(1+z_2(t))} - \frac{7}{5} z_5(t) + \zeta_5(t) \right] \Delta t \\
z_6(k+1) = z_6(k) + \left[\frac{(4|s|-16)}{15} + \frac{(8-2|s|)}{15(1+z_1(t))} + \frac{(8-2|s|)}{15(1+z_2(t))} - \frac{8}{5} z_6(t) + \zeta_6(t) \right] \Delta t \\
z_7(k+1) = z_7(k) + \left[\frac{(18-4|s|)}{15} + \frac{(2|s|-9)}{15(1+z_1(t))} + \frac{(2|s|-9)}{15(1+z_2(t))} - \frac{9}{5} z_7(t) + \zeta_7(t) \right] \Delta t \\
z_8(k+1) = z_8(k) + \left[\frac{2z_1(t)}{15(1+z_1(t))} - \frac{2z_2(t)}{15(1+z_2(t))} - \frac{2z_6(t)}{5(1+z_6(t))} + \frac{2z_{10}(t)}{5(1+z_{10}(t))} + \frac{3z_{12}(t)}{5(1+z_{12}(t))} \right. \\
\left. + \frac{z_{15}(t)}{5(1+z_{15}(t))} - \frac{z_{16}(t)}{5(1+z_{16}(t))} - 2z_8(t) + \zeta_8(t) \right] \Delta t \\
z_9(k+1) = z_9(k) + \left[- \frac{z_1(t)}{5(1+z_1(t))} - \frac{z_2(t)}{5(1+z_2(t))} - \frac{3z_6(t)}{5(1+z_6(t))} - \frac{11}{5} z_9(t) + \zeta_9(t) \right] \Delta t \\
z_{10}(k+1) = z_{10}(k) + \left[\frac{dz_{10}(t)}{dt} = \frac{3z_{12}(t)}{5(1+z_{12}(t))} - \frac{12}{5} z_{10}(t) + \zeta_{10}(t) \right] \Delta t \\
z_{11}(k+1) = z_{11}(k) + \left[\frac{dz_{11}(t)}{dt} = \frac{z_{12}(t)}{4(1+z_{12}(t))} - \frac{13}{5} z_{11}(t) + \zeta_{11}(t) \right] \Delta t \\
z_{12}(k+1) = z_{12}(k) + \left[\frac{dz_{12}(t)}{dt} = \frac{2z_{15}(t)}{5(1+z_{15}(t))} - \frac{2z_{16}(t)}{5(1+z_{16}(t))} - \frac{14}{5} z_{12}(t) + \zeta_{12}(t) \right] \Delta t \\
z_{13}(k+1) = z_{13}(k) + \left[- \frac{z_{15}(t)}{5(1+z_{15}(t))} - \frac{19z_{16}(t)}{5(1+z_{16}(t))} - 5z_{13}(t) + \zeta_{13}(t) \right] \Delta t \\
z_{14}(k+1) = z_{14}(k) + \left[- \frac{4z_{10}(t)}{5(1+z_{10}(t))} - \frac{4z_{12}(t)}{5(1+z_{12}(t))} - \frac{16}{5} z_{14}(t) + \zeta_{14}(t) \right] \Delta t \\
z_{15}(k+1) = z_{15}(k) + \left[\frac{z_{16}(t)}{10(1+z_{16}(t))} - \frac{7}{2} z_{15}(t) + \zeta_{15}(t) \right] \Delta t \\
z_{16}(k+1) = z_{16}(k) + \left[\frac{z_{15}(t)}{10(1+z_{15}(t))} - \frac{7}{2} z_{16}(t) + \zeta_{16}(t) \right] \Delta t \\
z_{17}(k+1) = z_{17}(k) + \left[- \frac{z_{15}(t)}{10(1+z_{15}(t))} + \frac{z_{16}(t)}{10(1+z_{16}(t))} - \frac{19}{5} z_{17}(t) + \zeta_{17}(t) \right] \Delta t \\
z_{18}(k+1) = z_{18}(k) + \left[- \frac{z_{15}(t)}{10(1+z_{15}(t))} + \frac{z_{16}(t)}{10(1+z_{16}(t))} - \frac{z_{17}(t)}{5(1+z_{17}(t))} - 4z_{18}(t) + \zeta_{18}(t) \right] \Delta t
\end{cases}
\quad (S2)
$$

Where $Z(k)$ corresponds to the vector $Z(t)$ at the time instant $k\Delta t$. The Jacobian matrix of Eq.(S2) is denoted as $J = \left.\frac{\partial f(Z(k);S)}{\partial Z}\right|_{Z=\overline{Z}}$, with

$$J = e^{\Delta t \bullet A} \qquad (S3)$$

By taking $\Delta t = 1$, eight distinct eigenvalues can be obtained from Eq.(S3). The largest eigenvalue satisfies $0.68^{|s|} \to 1$ when $s \to 0$, which is corresponding to that the largest eigenvalue of differential system Eq.(S1) approaches 0 from the left-hand side when $s \to 0$. Thus, the equilibrium point $\overline{Z}$ is stable if $s \in (0,1]$. The special parameter $s = 0$ is considered as the bifurcation point, where the system undergoes a qualitative transition.

From dynamical system viewpoint, a bifurcation point at which the system loses the stability of an equilibrium is the tipping point. The special parameter value " $s = 0$ " is the bifurcation point of the parameter in our model (the 18-node network), at which there is a bifurcation occurring, that is, when $s < 0$, real parts of all the eigenvalues for the Jacobian matrix of Eq. (S1) at the equilibrium are negative, which means that originally the equilibrium point $\overline{Z} = (\overline{z_1}, \overline{z_2}, \overline{z_3}, ..., \overline{z_{18}}) = (0,0,0,...,0)$ is stable. When $s \to 0$, the largest real part (λ) among the all eigenvalues approaches 0 and then becomes positive after passing $s = 0$, which means that the original stable equilibrium $\overline{Z} = (\overline{z_1}, \overline{z_2}, \overline{z_3}, ..., \overline{z_{18}})$ becomes unstable and further bifurcates into a qualitatively different periodic solution (actually, a Hopf bifurcation occurs). Such bifurcation phenomenon stands for a phase change of the system. According to the theoretical model Eq.(S2), with parameter varying from -0.5 to 0.15, the numerical simulation dataset of the 18-nodes expressions was generated from the network.

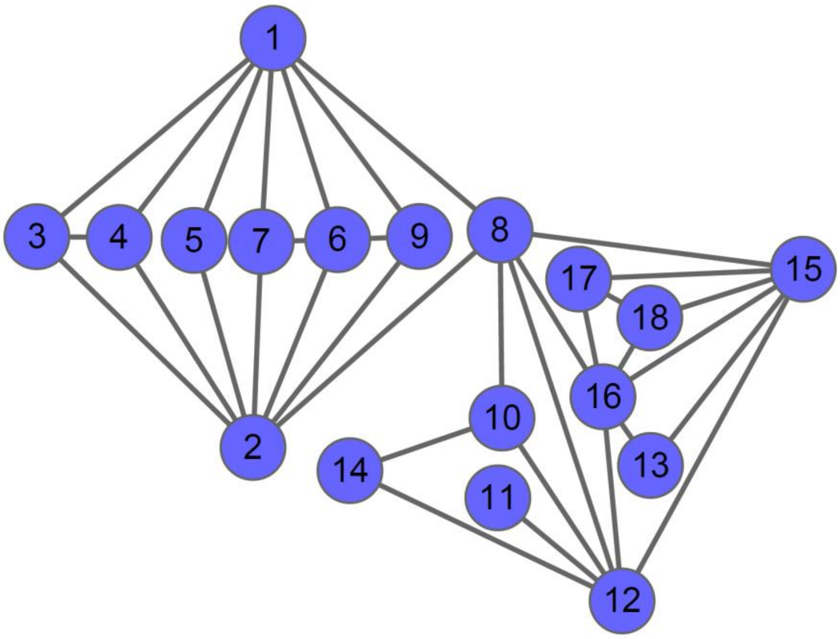

Figure S1. An 18-node regulatory network, from which the numerical simulation is generated. Such regulatory network represented in Michaelis-Menten form is described by the stochastic differential equations Eq. (S1)

We analyzed the stability and robustness of the proposed method under different levels of data noise. As depicted in Figure S2, the DNRS index accurately indicates the early warning signals of critical transition when the data noise strength σ varies. Therefore, the noise strength within a range barely affects the evolution tendency of the signal curve (e.g., an abrupt increase when approaching the tipping point).

In addition, we have also analyzed the DNRS score curve with a new parameter value $s = 0$ instead of the original value $s = -0.001$ (Figure S3). It is seen that the tendency of the new DNRS score curve can accurately indicate the critical transition of the network system with similar tendency, which demonstrate that the DNRS score still works when the parameter $s = -0.001$ representing the pre-transition state is replaced by a special parametric value $s = 0$.

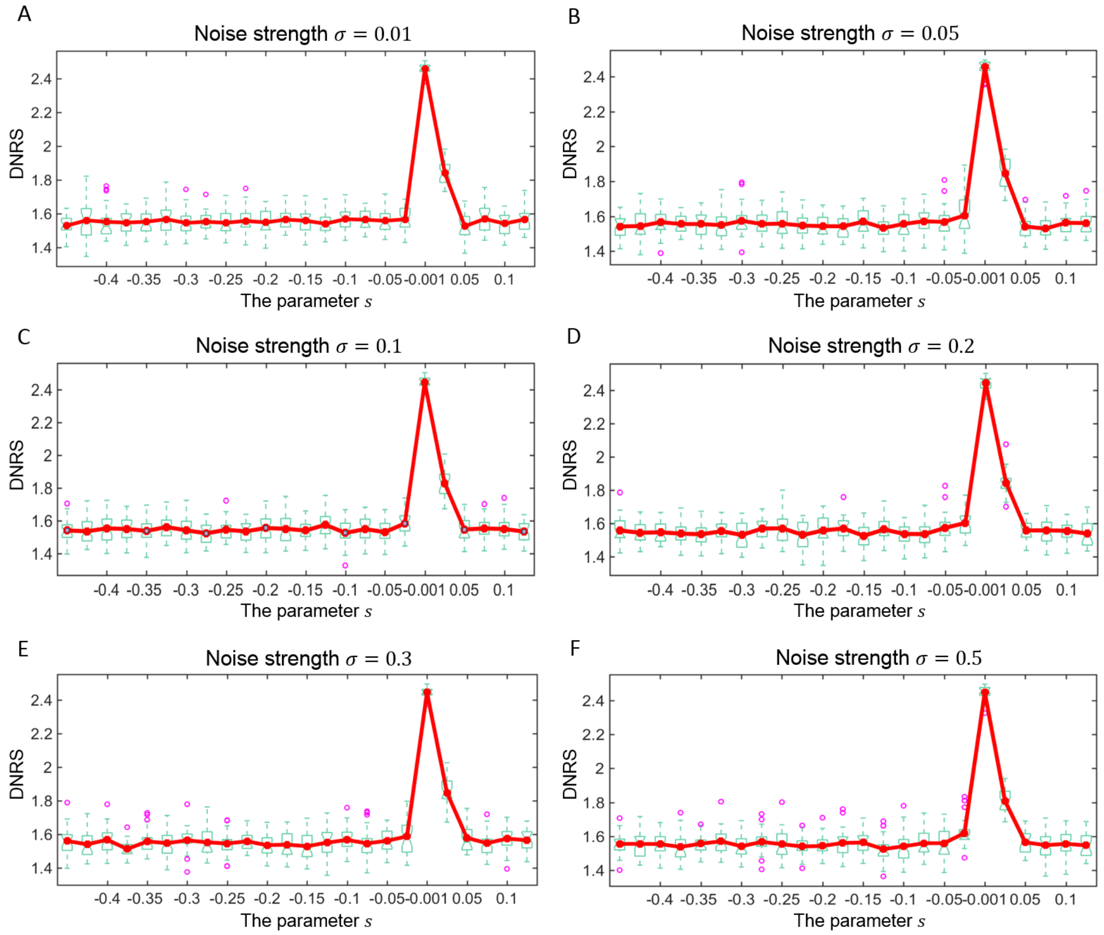

Figure S2: The critical signals under different noise strength σ. Based on the numerical simulation dataset generated from the Eq.(S1) of the revised Supplementary Material, we have analyzed the early warning signals to the critical transition of the network system with different noise strength. (A) the DNRS curve for noise strength $\delta = 0.01$. (B) the DNRS curve for noise strength $\delta = 0.05$. (C) the DNRS curve for noise strength $\delta = 0.1$. (D) the DNRS curve for noise strength $\delta = 0.2$. (E) the DNRS curve for noise strength $\delta = 0.3$. (F) the DNRS curve for noise strength $\delta = 0.5$.

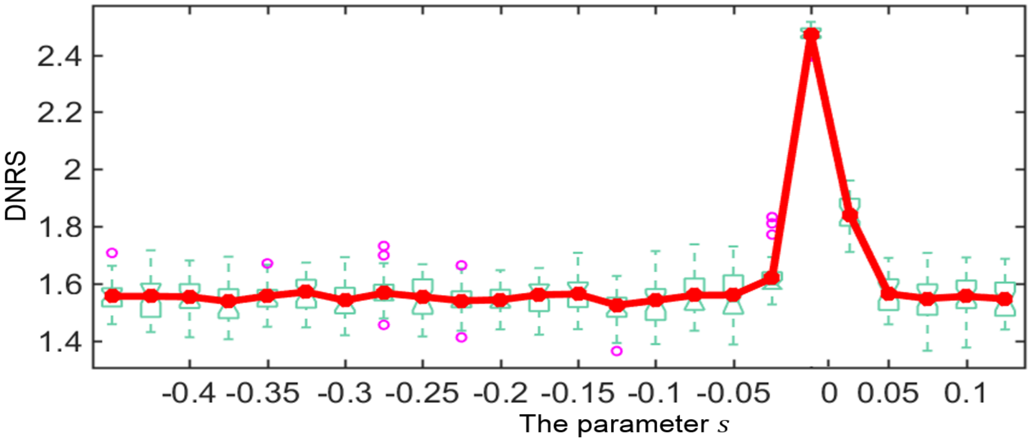

Figure S3: the tendency of the DNRS score curve with a new parameter value $s = 0$.

B. Description of the six datasets

To validate the effectiveness of the proposed computational method, we applied the DNRS method to six real biological datasets, including three tumor datasets (COAD, KIRC, and LUAD) from The Cancer Genome Atlas (TCGA) database (http://cancergenome.nih.gov) and three scRNA-Seq datasets of embryonic development (EBC-to-MHF data, hESC-to-DEC data, and hESC-to-neuron data) from Gene Expression Omnibus (GEO) database (http://www.ncbi.nlm.nih.gov/geo/). The detailed description and sources of the datasets are listed as follows.

The TCGA-COAD dataset includes 420 tumor samples and 41 tumor-adjacent samples. According to corresponding clinical information of TCGA, tumor samples are divided into stage I (72 samples), stage II (165 samples), stage III (121 samples), and stage IV (62 samples). The gene expression profiling data were downloaded from https://portal.gdc.cancer.gov/projects/TCGA-COAD.

The TCGA-KIRC dataset consists of 353 tumor samples and 72 tumor-adjacent samples. Based on corresponding clinical information of TCGA, tumor samples are grouped into stage I (198 samples), stage II (41 samples), stage III (64 samples), and stage IV (50 samples). The gene expression profiling data could be derived from https://portal.gdc.cancer.gov/projects/TCGA-KIRC.

The TCGA-LUAD dataset is composed of 421 tumor samples and 58 tumor-adjacent samples. On the basis of tumor samples are divided into stage IA (106 samples), stage IB (124 samples), stage IIA (39 samples), stage IIB (59 samples), stage IIIA (62 samples), stage IIIB (10 samples), and stage IV (21 samples). The gene expression profiling data were obtained from https://portal.gdc.cancer.gov/projects/TCGA-LUAD.

EBC-to-MHF data [3] describes the development of epithelial basal cells to mouse hair follicle. This dataset includes 1614 single cells in total. Cell numbers are also taken at E11.5 (94 cells), E12 (280 cells), E13 (276 cells), E13.5 (189 cells), E14 (183 cells), E15 (362 cells), and E17 (282 cells). The normalized data were available from GEO under accession number GSE147372.

hESC-to-DEC data [4] is generated from a time-course of human embryonic stem cells (hESC) from the pluripotent state though a mesendoderm intermediate to definitive endoderm cells (DEC). There are 758 single cells in total. Cell numbers are taken and profiled by scRNA-seq at 0 h (92 cells), 12 h (102 cells), 24 h (66 cells), 36 h (172 cells), 72 h (138 cells), and 96 h (188 cells). The normalized data were derived from GEO under accession number GSE75748.

hESC-to-neuron data [5] presents the differentiation of progenitor cells into neuronal cells. There are 483 single-cell samples in total. a total of 2684 cells with 6 time points are obtained during embryonic development, which includes 40 single cells at embryonic day 0, 504 at day 12, 278 at day 19, 595 at day 26, 502 at day 40, and 765 at day 54. The normalized data were downloaded from GEO under accession number GSE86977.

C. The derivation for direction discrimination index *w*

For the discrete variable $\vec{X}$ and $\vec{Y}$, the mutual information (MI) can be denoted as follows.

$$MI(\vec{X},\vec{Y}) = \sum_{x\in\vec{X}}\sum_{y\in\vec{Y}} p(x,y)\log\frac{p(x,y)}{p(x)p(y)} = -\sum_{x\in\vec{X}} p(x)\log p(x) - \sum_{y\in\vec{Y}} p(y)\log p(y) + \sum_{x\in\vec{X}}\sum_{y\in\vec{Y}} p(x,y)\log p(x,y) = H(\vec{X}) + H(\vec{Y}) - H(\vec{X},\vec{Y}) \quad \text{(S4)}$$

where $H(\vec{X})$ and $H(\vec{Y})$ are the entropies of the variable $\vec{X}$ and $\vec{Y}$, respectively. The $H(\vec{X},\vec{Y})$ is the joint entropy of $\vec{X}$ and $\vec{Y}$. The entropy can be estimated with Gaussian kernel probability density estimator [6] as follows.

$$P(x_i) = \frac{1}{N}\sum_{i=1}^{N}\frac{1}{(2\pi)^{\frac{n}{2}}|C|^{\frac{n}{2}}}\exp\left(-\frac{1}{2}(x_j - x_i)^T C^{-1}(x_j - x_i)\right) \quad \text{(S5)}$$

where C is the covariance matrix of variable $\vec{X}$, the $|C|$ is he determinant of matrix C, N is the number of samples and n is the number of variables in C. With Eq. (S5), we can the entropy of variable $\vec{X}$ as follows.

$$H(\vec{X}) = -\sum_{x\in\vec{X}} p(x)\log p(x) = \frac{1}{2}\log(2\pi e)^n|C| \quad \text{(S6)}$$

Therefore, with Eq. (S6), the $MI(\vec{X},\vec{Y})$ can be expressed follows.

$$MI(\vec{X},\vec{Y}) = H(\vec{X}) + H(\vec{Y}) - H(\vec{X},\vec{Y}) = -\frac{1}{2}\log\frac{|C(\vec{X},\vec{Y})|}{|C(\vec{X})||C(Y)|} \quad \text{(S7)}$$

Note that

$$|C(\vec{X},\vec{Y})| = \begin{vmatrix} C(\vec{X}) & cov(\vec{X},\vec{Y}) \\ cov(\vec{X},\vec{Y}) & C(\vec{Y}) \end{vmatrix} = |C(\vec{X})||C(\vec{Y})| \begin{vmatrix} 1 & \frac{cov(\vec{X},\vec{Y})}{|C(\vec{X})|} \\ \frac{cov(\vec{X},\vec{Y})}{|C(\vec{Y})|} & 1 \end{vmatrix}$$

$$= |C(\vec{X})||C(\vec{Y})|\left(1 - \left(\frac{cov(\vec{X},\vec{Y})}{\sqrt{|C(\vec{X})||C(\vec{Y})|}}\right)^2\right) = |C(\vec{X})||C(\vec{Y})|\left(1 - PCC(\vec{X},\vec{Y})^2\right) \quad \text{(S8)}$$

where $PCC(\vec{X},\vec{Y})$ represents Pearson correlation coefficient between $\vec{X}$ and $\vec{Y}$.

With Eq. (S7) and Eq. (S8), the $MI(\vec{X},\vec{Y})$ can be simplified as below.

$$MI(\vec{X},\vec{Y}) = -\frac{1}{2}\log\left(1 - PCC(\vec{X},\vec{Y})^2\right) \quad \text{(S9)}$$

According to previous studies [7,8], the binomial distribution can be approximated by the Gaussian distribution. Therefore, if we assume the Gaussian distribution or binomial distribution for variables, with Eq. (S9), the Eq. (3) in main text can be expressed as follows.

$$w_{i,j} = \sum_{\hat{x}\in\vec{X}}\sum_{y\in\vec{Y}} p(\hat{x},y)\log\frac{p(\hat{x},y)}{p(\hat{x})p(y)} - \sum_{x\in\vec{X}}\sum_{y\in\vec{Y}} p(x,y)\log\frac{p(x,y)}{p(x)p(y)}$$

$$= MI\left(\vec{\tilde{X}},\vec{\tilde{Y}}\right) - MI\left(\vec{X},\vec{Y}\right) = -\frac{1}{2}\log\frac{1-PCC(\vec{\tilde{X}},\vec{\tilde{Y}})^2}{1-PCC(\vec{X},\vec{Y})^2} \quad \text{(S10)}$$

D. Description of identifying the critical point by the one-sample t-test

To quantify how well the DNRS recapitulates the abrupt transition, the one-sample t-test is applied to determine whether there is a statistical difference between the before-transition and pre-transition stages. The one-sample *t*-test statistics Z is defined below Eq.(S11) and then applied to determine whether the constant x is significantly different from the mean of an *n*-dimensional vector $X = (x_1, x_{2,\cdots,}x_n)$.

$$Z = \frac{mean(X)-x}{s/\sqrt{n}} \quad \text{(S11)}$$

where $mean(X)$ is expressed as the mean of the vector X and constant x is the standard deviation of the vector X. The P value related to index Z is derived from the t-distribution to assess the statistical difference between $mean(X)$ and x. There is statistically significant between $mean(X)$ and x if $P < 0.05$. In this study, if the time point $T = t\ (t > 2)$ is regarded as a critical point if the DNRS value $PR(t)$ should satisfy following two conditions: (i) $PR(t)$> $PR(t-1)$ and (ii) $PR(t)$ is a statistical difference ($P < 0.05$) from the mean of the vector X=($PR(1), PR(2), \cdots, PR(t-1)$). When the DNRS value $PR(T=2)$ meets the following requirements: (i) $PR(2)$> $PR(1)$ and (ii) $PR(2)$ is a significant difference ($P < 0.05$) from the mean of the vector X=($PR(1), PR(3)$), the time point $T = 2$ is considered as the critical point.

E. Validating the identified critical stage by the Kaplan-Meier log rank analysis

The proposed approach has been applied to three stage-course TCGA datasets, i.e., COAD, KIRC, and LUAD. There are three requirements for the dataset selection: 1, they are all stages datasets, which indicate the state of cancer; 2, both tumor and adjacent non-tumor samples are available in these datasets; 3, the corresponding clinical information is available in all these datasets so as to perform the survival analysis. For three tumor datasets, as presented in Figs 3G-3L of the main text, the proposed DNRS method successfully indicated the pre-transition stage before the lymph node metastasis (stage II) for colon adenocarcinoma (COAD) and kidney renal clear cell carcinoma (KIRC), and the pre-transition stage before the distant metastasis (stage IIIB) for lung adenocarcinoma (LUAD). To validate the identified critical stage,

survival times based on samples from before-transition and after-transition stage, respectively, were exhibited and compared using the Kaplan-Meier log rank analysis. Specifically, there are two criteria for validating the identified pre-deterioration stage: (1), the samples derived before the identified pre-deterioration stage are generally with higher survival rates than those derived after the identified pre-deterioration stage; (2), the survival time of samples from the identified pre-deterioration stage is significantly longer ($P<0.05$) than that of samples from the next stage (Figure S4). The following Figure S4 indicated that the survival time of after-transition samples is statistically longer than that of samples from before-transition stage. For COAD, as shown in Figure S5A, the survival curves indicate that the survival time of samples before stage II is significantly ($p = 0$) longer than that after stage II. In addition, the survival time of samples from stage II (the critical point) are significantly different ($p = 0.0413$) from that of stage III-IV samples (Figure S5D). When applied to KIRC, there is a statistical difference ($p = 0$) between the survival curves of samples before and after stage II (Figure S5B). Additionally, as illustrated in Figure S5E, the survival time of patients from stage II (the critical stage) is much longer than that after stage II. For LUAD, the survival curves before stage III (the critical stage) were significantly different ($p = 0.0242$) from the curves afte stage III (Figure S5C). These results demonstrate that the DNRS method could detect the early-warning signals of a critical transition of patient survival.

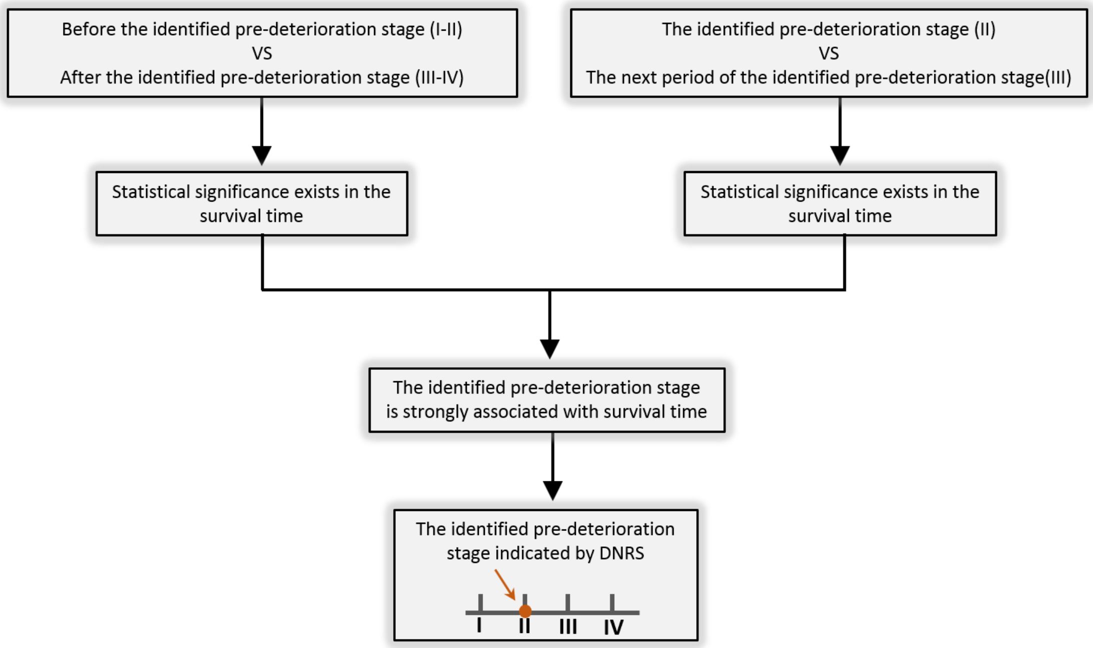

Figure S4: an illustrative process for validating the identified pre-deterioration stage.

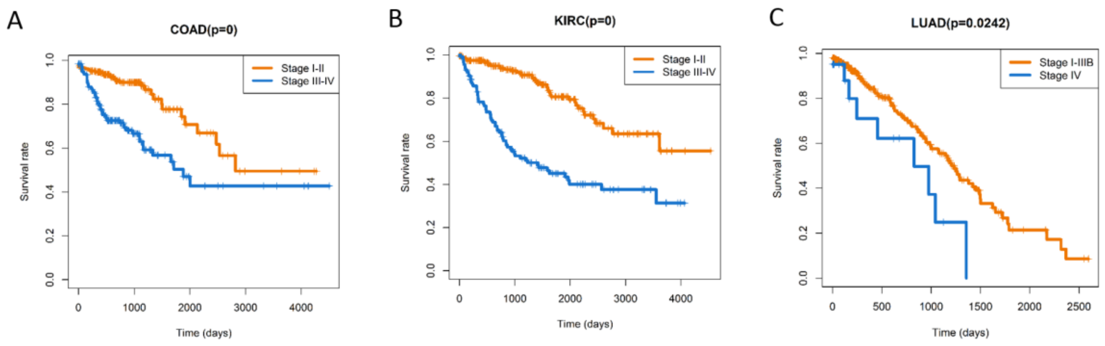

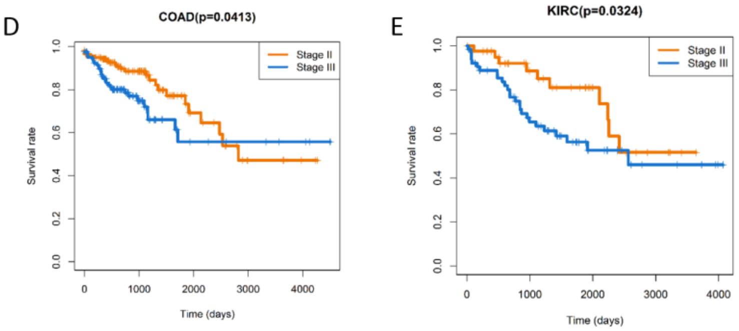

Tumor	The identified critical stage
COAD	Stage II
KIRC	Stage II
LUAD	stage IIIB

Figure S5: Survival analysis before and after the identified critical stage in three cancers: (A)&(D) COAD, (B)&(E) KIRC, and (C) LUAD.

F. The dynamical evolution of the regulatory networks for signaling genes

At identified tipping point, we selected the top 5% genes with the largest local DNRS as the signaling genes. The signaling genes were mapped into the protein-protein interaction (PPI) network, where the maximal connected subgraph was extracted to study the dynamical evolution of the regulatory network for signaling genes. For hESC-to-neuron data, the dynamical evolution of the regulatory network across all 5 time points was presented in Figure S6A, where an obvious change occurred in the network structure at day 26, indicating the cell fate transition of progenitor cells into neuronal cells at day 40 [5]. When applied to hESC-to-DEC data, there was a notable change in the network structure at 36 h (Figure S6B), implying the commitment to a definitive endoderm fate occurred after 36 h [4].

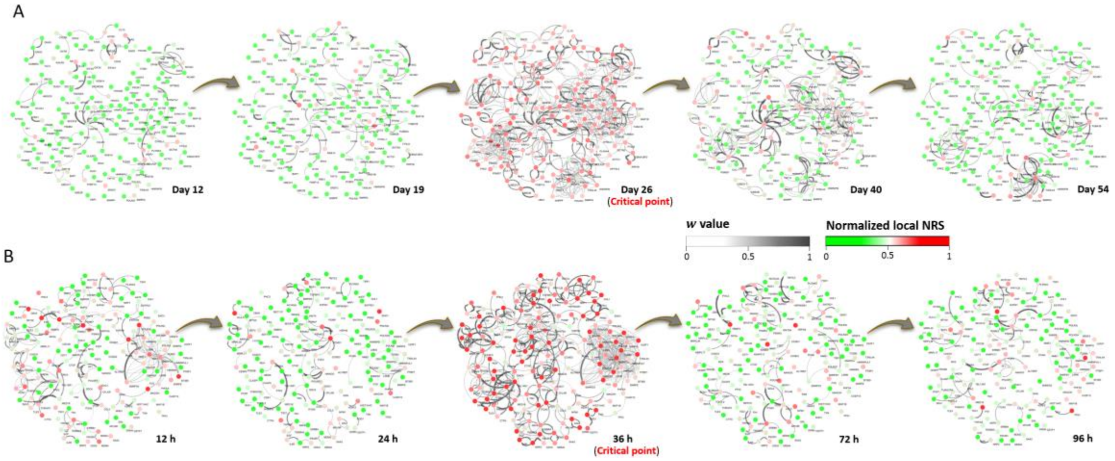

Figure S6: (A) The dynamical evolution of the regulatory network for hESC-to-neuron data. (B) The dynamical evolution of the regulatory network for hESC-to-DEC data.

G. Common signaling genes shared among different datasets

At identified critical stage, we selected the top 5% genes with the largest local DNRS as the signaling genes for further biological functional analysis. As shown in Figure S7A, the 24 common signaling genes (CSGs) were identified for human embryo development from two scRNA-seq datasets (hESC-to-DEC and hESC-to-neuron data). For COAD, KIRC, and LUAD, there were not only many intersections across the signaling genes in different cancers, but there existed close functional relationships among them (Figure S7B). It can be seen from Figure S7C there are 90 common signaling genes among these three tumors. GO analysis demonstrated that these common genes are enriched in biological processes associated with cancer progression, such as regulation of Wnt signaling pathway, regulation of MAP kinase activity, and Ras protein signal transduction (Figure S7D). Besides, through literature searching, as shown in Table S1, some common signaling genes in three different cancers have been reported to be closely associated with tumor progression, suggesting that these genes play important functions in the development and progression of cancer.

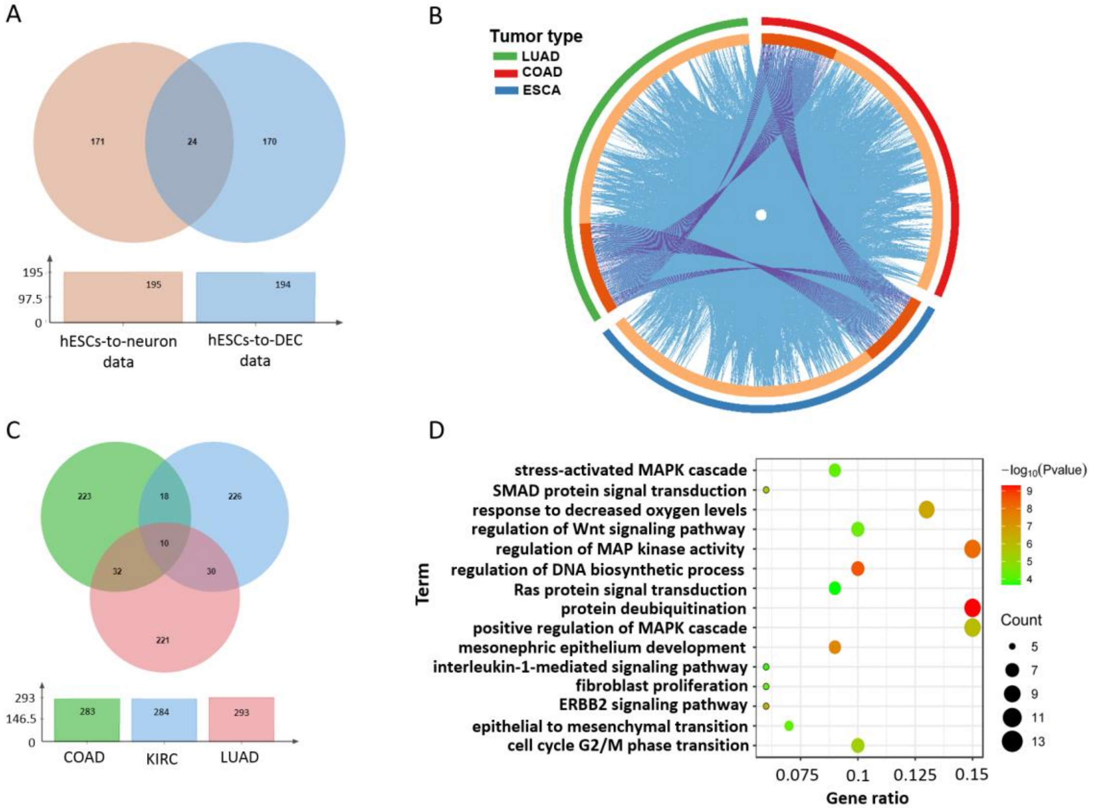

Figure S7: Common signaling genes shared between different datasets. (A) Common signaling genes between hESC-to-DEC and hESC-to-neuron data. (B) A large amount of overlap presents not only in the identical signaling genes among three tumors, but in their biological functions. The outer ring stands for different groups of DNRS-signaling genes, and the inner ring stands for their identical genes and functions. The identical genes are linked with each other by purple lines, and functions are depicted in blue lines. (C) Common signaling genes among COAD, KIRC, and LUAD. (D) Functional enrichment through GO analysis indicated that the 90 common signaling genes are enriched in biological processes associated with cancer progression.

Table S1. The information of important common signaling genes in three different cancers

Gene	Family	Relation with tumor development
HSPA8	chaperone	*HSPA8* plays a significant role in endothelial cells via PI3K-Akt pathway and increases cell survival by inhibiting the apoptotic program (Shiota et al., 2010).
DVL1	regulatory	*DVL1* with *APC* gene mediate a WNT5A-FZD2 signal to focal adhesions to regulate cell-substrate adhesions and migration (Matsumoto et al., 2010).
RPN2	enzyme	*RPN2* is a significant oncogene and plays a crucial role in cancer progression and drug resistance (Sun et al., 2020).

NEK2	enzyme	*NEK2* activation can lead to cancer cell proliferation when it is aberrantly regulated (Sankaran et al., 2006).
BCL2	regulatory	*BCL2* serves as a regulator of cancer invasion and metastasis (Um, 2016).
TP53	transcription factor	*TP53* is a transcription factor that mediates tumor suppressor responses in tumor development (Trinidad et al., 2013).
SMPD1	enzyme	*SMPD1* is an important enzyme in sphingolipid metabolism and plays key roles in apoptosis, development, and cancer (Perrotta et al., 2010).
SMAD7	regulatory	*SMAD7* can detect and regulate the tipping point of TGF-beta induced epithelial–mesenchymal transition (Jiang et al., 2020).

H. Several improvements of DNRS compared with the classical DNB method

Compared with the classical DNB method, there are several advantages of the DNRS method. First, the DNRS method can reconstructs a set of the time-specific directed network and captures the significant dynamic changes in gene associations during the progression of a biological process. Second, the DNRS method can effectively analyze scRNA-seq data, which usually suffers from heterogeneous, sparse, and noisy samples in contrast to conventional bulk RNA-seq information. For the hESC-to-neuron dataset (scRNA-seq data) shown as in Figure S8A and B, a significant increase ($P = 0.0027$) of the DNRS score is detected in day 26, but there is no abrupt increase in the cDNB score, that is, the proposed DNRS method can detect the critical transition point, while the classical DNB method fails. Third, the DNRS method not only detects general early-warning signals of the catastrophic transition, but provides a ranked gene list for the importance of genes, in which each gene has a specific weight (local DNRS score). Besides, the proposed DNRS method is characterized by strong robustness. When applied to TCGA-KIRC dataset with large noise (Figure S8C and D), the signal of critical transition from the DNRS method ($P = 1.592E - 04$) is more significant than that from the classical DNB method ($P = 0.014$).

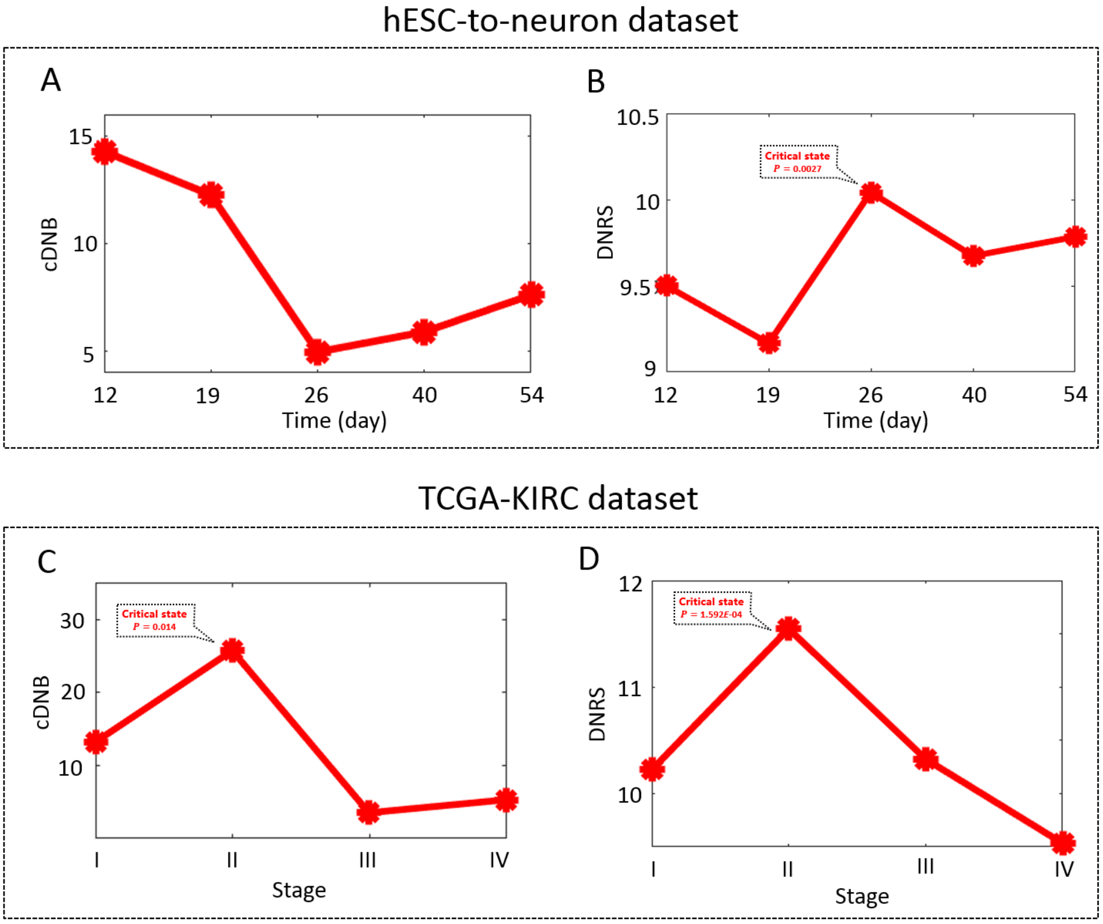

Figure 8: Comparison of dynamic changes performance between the proposed DNRS approach and classical DNB method. The performances of the two methods in scRNA-seq data for (A)-(B) hESC-to-neuron data. It is seen that there is no signal based on cDNB, while there is a significant increase (with P-value 0.0027) of DNRS. Comparison performance of dynamic changes in bulk RNA-seq data with large noise for (C)-(D) TCGA-KIRC dataset. The signal from DNRS (with P-value 1.592E-04) is much more significant than that from cDNB (with P-value 0.014).

I. Analysis of the different settings of the adjustable parameter Q

The EBC-to-MHF and TCGA-LUAD dataset have been employed to analyze the critical signals when the adjustable parameter Q ranges from the top 1% to 10%. It can be seen from Figure S9 that the DNRS index accurately indicates the tipping point with a similar tendency. Therefore, different settings of the adjustable parameter Q within a range (usually from the top 1% to 10%) do not affect the evolution tendency of the signal curve.

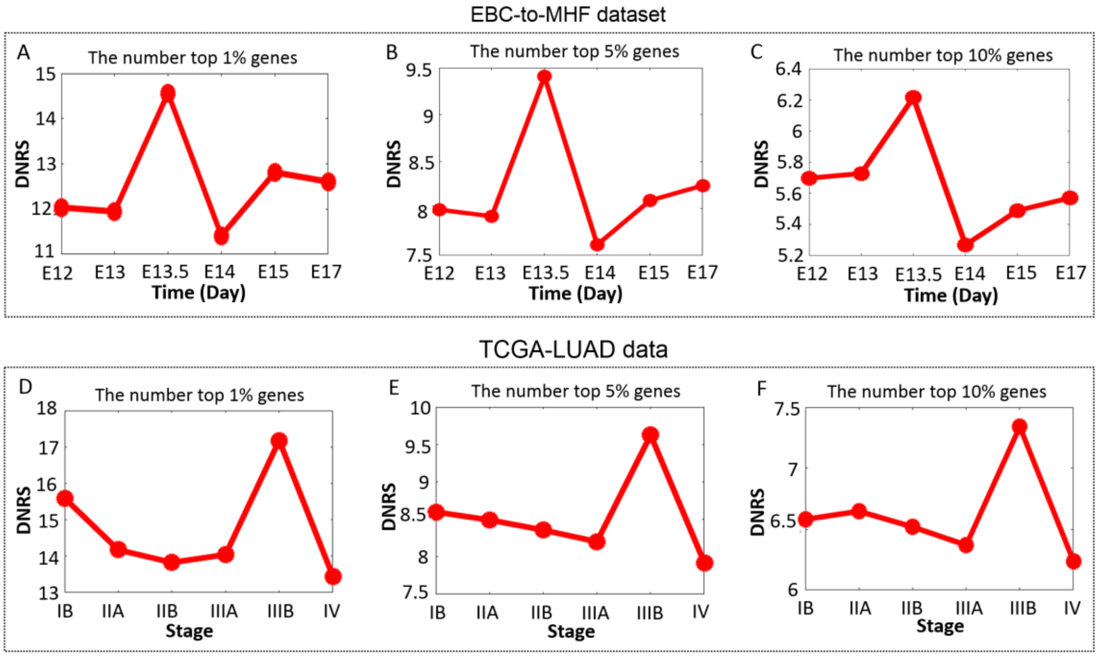

Figure S9: the critical signals of EBC-to-MHF and TCGA-LUAD dataset under different settings of the adjustable parameter Q. For the EBC-to-MHF dataset, the adjustable parameter Q is set as (A) the number of top 1% genes, (B) the number of top 5% genes, and (C) the number of top 10% genes, respectively. For the TCGA-LUAD dataset, the adjustable parameter Q is set as (D) the number of top 1% genes, (E) the number of top 5% genes, and (F) the number of top 10% genes, respectively.

References

1. Chen, L., Wang, R. & Zhang, X. Biomolecular Networks: Methods and Applications in Systems Biology, (John Wiley & Sons, Hoboken, New Jersey,2009).

2. Chen, L. & Aihara, K. Stability of genetic regulatory networks with time delay, IEEE Trans. Circuits Syst. I 49, 602–608(2002).

3. Morita R, Sanzen N, Sasaki H, Hayashi T, Umeda M, Yoshimura M, et al. Tracing the origin of hair follicle stem cells. Nature (London). 2021;594(7864):547-52.

4. Chu L, Leng N, Zhang J, Hou Z, Mamott D, Vereide DT, et al. Single-cell RNA-seq reveals novel regulators of human embryonic stem cell differentiation to definitive endoderm. Genome Biology. 2016;17(1):173-.

5. Yao Z, Mich JK, Ku S, Menon V, Krostag A, Martinez RA, et al. A single-cell roadmap of lineage bifurcation in human ESC models of embryonic brain development. Cell stem cell. 2017;20(1):120-34.

6. Zhang X, Zhao X, He K, Lu L, Cao Y, Liu J, et al. Inferring gene regulatory networks from gene expression data by path consistency algorithm based on conditional mutual information. BIOINFORMATICS. 2012;28(1):98-104.

7. Malikic S, Jahn K, Kuipers J, Sahinalp SC, Beerenwinkel N. Integrative inference of subclonal tumour evolution from single-cell and bulk sequencing data. Nature communications. 2019;10(1):2750-12.

8. Burdukiewicz M, Rödiger S, Sobczyk P, Menschikowski M, Schierack P, Mackiewicz P. Methods for comparing multiple digital PCR experiments. Biomolecular detection and quantification. 2016;9(C):14-9.

9. Shiota M, Kusakabe H, Izumi Y, Hikita Y, Nakao T, Funae Y, et al. Heat Shock Cognate Protein 70 Is Essential for Akt Signaling in Endothelial Function. Arteriosclerosis, thrombosis, and vascular biology. 2010;30(3):491-7.

10. Matsumoto S, Fumoto K, Okamoto T, Kaibuchi K, Kikuchi A. Binding of APC and dishevelled mediates Wnt5a-regulated focal adhesion dynamics in migrating cells. The EMBO journal. 2010;29(7):1192-204.

11. Sun J, Ma Q, Li B, Wang C, Mo L, Zhang X, et al. RPN2 is targeted by miR-181c and mediates glioma progression and temozolomide sensitivity via the wnt/β-catenin signaling pathway. Cell death & disease. 2020;11(10):890.

12. Sankaran S, Parvin JD. Centrosome function in normal and tumor cells. Journal of cellular biochemistry. 2006;99(5):1240-50.

13. Um H. Bcl-2 family proteins as regulators of cancer cell invasion and metastasis: A review focusing on mitochondrial respiration and reactive oxygen species. Oncotarget. 2016;2015;7(5):5193-203.

14. Trinidad A, Muller PJ, Cuellar J, Klejnot M, Nobis M, Valpuesta J, et al. Interaction of p53 with the CCT Complex Promotes Protein Folding and Wild-Type p53 Activity. Molecular cell. 2013;50(6):805-17.

15. Perrotta C, Bizzozero L, Cazzato D, Morlacchi S, Assi E, Simbari F, et al. Syntaxin 4 Is Required for Acid Sphingomyelinase Activity and Apoptotic Function. The Journal of biological chemistry. 2010;285(51):40240-51.

16. Jiang Z, Lu L, Liu Y, Zhang S, Li S, Wang G, et al. SMAD7 and SERPINE1 as novel dynamic network biomarkers detect and regulate the tipping point of TGF-beta induced EMT. Science bulletin (Beijing). 2020;65(10):842-53.
